# Evidence of ethnic variations in the relationships between routinely recorded clinical factors and T2D: a systematic review and meta-analysis

**DOI:** 10.1038/s41366-025-01848-9

**Published:** 2025-08-11

**Authors:** Binur Orazumbekova, Tooba Hamdani, Sam Hodgson, Miriam Samuel, Daniel Stow, Marie Spreckley, Sarah Finer, Moneeza K. Siddiqui, Rohini Mathur

**Affiliations:** 1 Wolfson Institute of Population Health, Queen Mary University of London, London, UK; 2 MRC Epidemiology Unit, University of Cambridge School of Clinical Medicine, Cambridge, UK

**Keywords:** Risk factors, Epidemiology, Type 2 diabetes, Obesity, Endocrine system and metabolic diseases

## Abstract

**Background:**

Evidence on ethnic differences in factors associated with type 2 diabetes (T2D) is mixed. We aimed to systematically review evidence on ethnic variations in the relationships between routinely recorded demographic and clinical factors and T2D.

**Methods:**

We searched Medline Complete and Embase for observational studies published between 1990 and 2023 investigating ethnic differences in factors routinely recorded in clinical encounters associated with T2D. We used random and fixed-effects meta-analysis to quantitatively summarise effect sizes across studies where possible. Risk of bias and study quality were assessed using the Newcastle-Ottawa Scale and Joanna Briggs Institute tool. PROSPERO registration: CRD42023394148.

**Findings:**

Searches identified 10,694 studies, of which, 54 (*n* = 10 332,949 individuals) were eligible for inclusion, including 12 suitable for meta-analysis. Included studies reported ethnic differences in age at T2D diagnosis, anthropometric measures, and factors associated with women’s health. Compared to individuals of White ethnicity, people of diverse ethnic backgrounds had 2-4-fold higher incidence and prevalence of T2D and younger age of onset. Waist-to-hip ratio (WHR) was a better discriminator of T2D across all ethnic groups compared to body mass index (BMI). While the association between overweight/obese BMI and T2D was strongest for people of White ethnicity (OR 4.85 CI 3.53–6.68) followed by Black (OR 3.27 CI 2.48–4.30) and East Asian ethnicities (OR 3.06 CI 2.29–4.16), the association between WHR and T2D was strongest for people of Black (OR 2.74, CI 2.22–3.39) than for White ethnicities (OR 2.51, CI 2.30–2.74). Included studies highlighted the emerging importance of women-health-associated factors such as index of parity, birth weight and breastfeeding, especially among women of diverse ethnicities.

**Conclusion:**

Ratio measures of central adiposity may better identify T2D in ethnically diverse populations than measures of overall adiposity. Sex-specific factors must be considered when assessing T2D risk.

**Funding:**

Wellcome Trust Grant 218584/Z/19/Z.

## Introduction

Type 2 diabetes (T2D) is a global public health problem, which, as of 2021, affects over 537 million people worldwide [1]. This number is expected to increase to 783 million by 2045 [1] along with associated healthcare expenditure [2]. The prevalence and incidence of T2D varies by ethnicity [3–5]: people of Asian ethnicity [3, 6, 7] especially South Asian [3, 8], Black and Arab ethnicities [9], and individuals of other ethnic backgrounds [6, 7] have at least a two to fourfold risk of developing T2D compared to people of White ethnicity. People of these ethnicities are also at a greater risk of developing diabetes-related micro- and macro-vascular complications [10].

Previous research suggests the pathophysiology of T2D differs across ethnicities; While insulin resistance is a major driver of diabetes in White populations, ß-cell dysfunction plays an important role in people of South Asian ethnicity and lower hepatic insulin clearance in people of Black ethnicity [11, 12]. There is a growing body of literature indicating people of the above-mentioned ethnic backgrounds develop T2D at younger ages compared to people of White ethnicity [9, 13, 14]. Along with this, the proportion of individuals who develop T2D within normal body weight ranges (BMI 18.5–24.9 kg/m2) is higher among individuals of South Asian, Black, Hispanic and East Asian ethnic backgrounds compared to people of White ethnicity [9, 15]. Current guidelines for the prevention of T2D are based on evidence largely derived from White European populations and are limited to decreasing BMI for people with overweight and obesity for individuals of Asian ethnic background, identifying diabetes risk at earlier ages [16, 17]. However, the differing causal mechanisms for T2D are still poorly understood and current guidelines may be inadequate for managing T2D and its longer-term sequelae in diverse ethnic populations. While several systematic reviews and meta-analyses have described key factors associated with T2D [18–20], none have yet focussed on systematically comparing these across ethnic groups.

In this exploratory study, to inform clinical risk prediction and facilitate the translation of routinely available health data into practice, making it more inclusive and effective across diverse ethnic groups, and addressing current gaps in T2D prevention and management, we systematically reviewed published evidence on ethnic differences in the relationships between demographic and clinical factors routinely captured in clinical care settings and type 2 diabetes.

## Methods

We registered this systematic review with PROSPERO (registration number CRD42023394148). This study was funded by the Wellcome Trust Grant, reference 218584/Z/19/Z. The funder of the study had no role in study design, data collection, data analysis, data interpretation, or writing of the report.

### Data sources and search strategy

We searched Medline Complete and Embase databases according to the Preferred Reporting Items for Systematic Reviews and Meta-Analyses (PRISMA) guidelines. To ensure coverage of all potentially eligible studies, we prepared a list of T2D- and ethnicity-related Medical Subject Headings (MeSH) and keywords (Table S1).

### Selection process and data extraction

Study eligibility was assessed by two independent reviewers (BO and TH). We retrieved and merged search results from Medline Complete and Embase databases using Endnote reference management software. We removed duplicates and exported a list of unique studies to Rayyan reference management software [21]. The screening stage was blinded to both reviewers. After screening, conflicting results were discussed with the research team, following which we assessed full texts for eligibility. We contacted the authors of one paper without full text available to request access.

We included studies that met the following eligibility criteria: Studies published in English between 1990 and 2023, studies with an observational design (cross-sectional, case-control, cohort studies) focusing on the association between routinely collected demographic factors (age, sex and ethnicity) and clinical factors with T2D; studies which compared two or more ethnic groups, and studies where the outcome was T2D. As this was an exploratory systematic review, we did not predefine an exhaustive list of risk factors to allow for a comprehensive synthesis of the available evidence. We excluded studies that only reported results for one ethnic group. By doing so we focussed our systematic review on studies with the explicit aim of comparing effects across ethnicities. We excluded studies where the outcome was another type of diabetes (e.g. Type 1 diabetes, gestational diabetes), as well as prediabetes, impaired fasting glucose, impaired glucose tolerance and insulin resistance. We also excluded studies restricted to populations with specific health conditions or receiving specific treatments (e.g. people with serious mental illness, cancer, etc.); studies focusing on factors not routinely available in clinical records, including, but not limited to genetics, lifestyle factors (physical activity, alcohol consumption, sleep duration, smoking status), and environmental exposures (e.g., air pollution), or studies with small sample per ethnic group, which could be underpowered to find clinically informative associations (total *n* < 200 or fewer than 50 subjects per ethnic group).

### Risk of bias assessment

We used the Newcastle-Ottawa Scale (NOS) [22] and Joanna Briggs Institute tool (JBI) [23, 24] to assess the risk of bias. Two reviewers conducted the assessments independently and disagreements were discussed with a third senior reviewer. The risk of bias for each study was assigned as low, moderate or high risk.

### Data analysis

We conducted a narrative synthesis structured around the association between risk factors of interest and T2D across ethnicities and, if available, by age and sex. In studies reporting comparable associations, effect sizes and statistical significance were synthesised for each risk factor by ethnic group, as outlined by guidance on the conduct of narrative synthesis for systematic reviews [25]. Due to a lack of standardised approaches to grouping ethnicities, we aggregated findings into broad ethnic groups, as detailed in Table S2. This categorization facilitated the summarization and reporting of more generalisable findings. For example, individuals of Pakistani, Bangladeshi and Indian ethnicities were groups as South Asian. However, if the effect of risk factors was compared across single ethnicities, the ethnic categorization as reported by the original study was used.

Associations between routinely recorded clinical factors and T2D were meta-analysed and compared across ethnic groups when two or more studies reported on the same routinely collected factor(s). Given the heterogeneity in study designs and population characteristics (location, age groups, sex) random effect meta-analysis was used where more than two studies were included in meta-analysis, while fixed effects model meta-analysis was used where only two studies were eligible [26]. The heterogeneity between studies was quantified by I^2^ statistics, which ranges between 0% and 100%—greater indicating a higher between-study variability [26]. Where possible, we have provided sex-stratified meta-analysis estimates. Funnel plots were plotted to assess publication bias. Analysis was conducted using the *meta* package in R [27] and the *metan* package in Stata 15 [28].

## Results

**Fig. 1:**
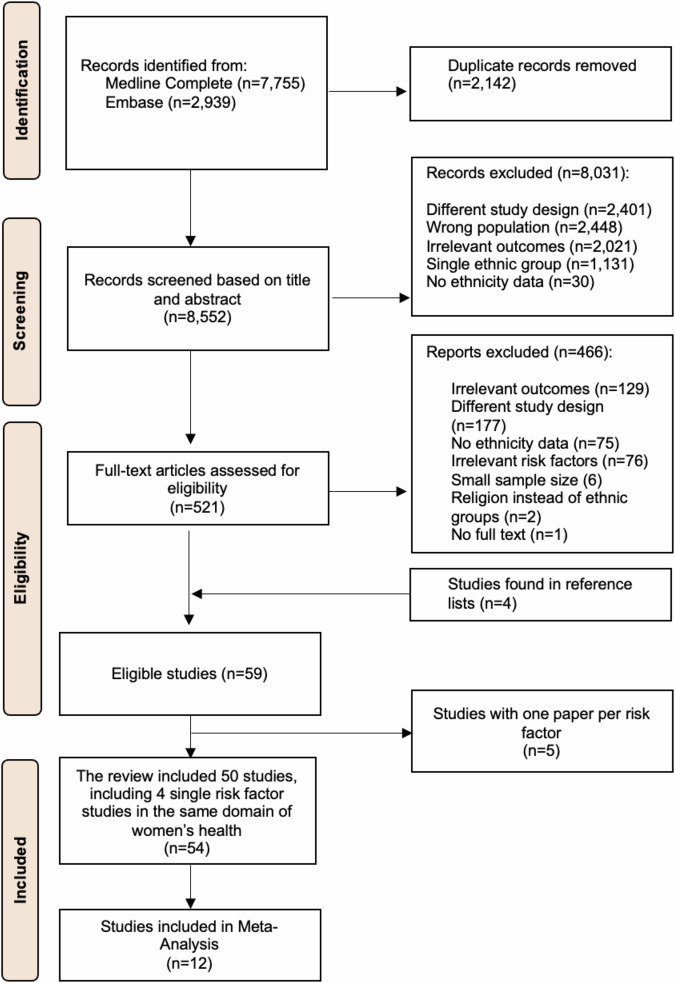
PRISMA flow chart.

The study selection process is represented in the PRISMA flow chart (Fig. 1). We identified 10,694 papers from two databases. After removing duplicates, 8552 titles and abstracts were screened, of which 8031 studies did not meet eligibility criteria. We reviewed the full texts of 521 articles and identified 59 eligible studies, including four from the reference lists of selected papers. Five risk factors were only reported in one study. Results from these studies are reported in the supplementary materials (Table S3). Several risk factors related to women’s health (pregnancy index, breastfeeding, birth weight, and age at menarche) were each reported in individual studies. Therefore, they are discussed together in the main text within the same paragraph for coherence, leaving 54 eligible, full-text papers. We described and narratively summarized the 54 included studies; 12 studies were included in the meta-analysis of BMI (*n* = 4) for OR, 2 for HR and 2 for RR, WHR (*n* = 2) and family history of diabetes (FHD) (*n* = 2).

### Study characteristics and risk of bias

Out of 54 studies, there were 29 cross-sectional, 21 cohort and 4 case-control studies coming from 15 countries and several combined populations from multiple countries, with an overall number of participants of 10,337,613. The main ethnic groups included in the studies were South Asian, Southeast Asian, Black, East Asian, White, Hispanic and others (Table S2). Full study characteristics are shown in Table 1.

**Table 1:** Study characteristics.

N	Author, Year, Title	Ethnic groups	Sample size, total and per each group	Number of cases (total, per ethnic group)	Data source (country)	Participants’ characteristics	Follow-up (years)	Risk factors	Diagnostic method of T2D
**Cross-sectional studies**									
**1**	**Alperet, 2016** [37]	Chinese, Malay, Asian-Indians	Total: 14,815. Chinese: 9012 Malay: 3130 Asian-Indians: 2,673	Chinese: 481 (5.34%) Malay: 299 (9.55%) Asian-Indians: 239 (8.94%)	Data from Singapore national Health Surveys conducted in 1992, 1998, 2004 and 2010 years (Singapore)	,Individuals aged 18–69 years (men and women). Median age - 38 years, 46.9%—males. Median age at T2D diagnosis, (IQR) for UDM: 49, (42–59)	–	BMI, WC, WHR, WHTR (adjusted for age and sex)	WHO criteria for UDM: FPG levels of ≥7.0 mmol/L or a 2-h post-load glucose level of ≥11.1 mmol/L
**2**	**De Koning, 2010** [36]	Aboriginal, African, South Asian, European, Latin American	Total: 22,293 Aboriginal: 12.1% (2697), African: 6.5% (1458), South Asian: 20.3% (4,535), European: 52.9% (11,795), Latin American: 8.1% (1808)	Total: 14% (3112) Aboriginal: 9.9% (267), African: 18.2% (265), South Asian: 14.1% (640), European: 14.5% (1712), Latin American: 12.6% (228).	The EpiDREAM cohort from the DREAM trial - a large, international, multi-centre, randomised and double-blind controlled trial (international cohort (21 countries))	60% women, 40% men. Mean age was 52.2 (11.4). 44.3% (9861) were smokers. 28.5% (5054) were post-menopausal women. Mean age of T2D diagnosis: Aboriginals: 52.0 (11.6), Africans: 53.9 (11.0), South Asians: 44.7 (9.2), Europeans: 55.1 (10.8), Latin Americans: 51.3 (11.4)	–	BMI, HC, BMI + WC, BMI + WHR	FPG ≥ 7.0 mmol/l (126 mg/dl) or a 2 h glucose ≥ 11.1 mmol/l (200 mg/dl)
**3**	**Hardy 2017** [31]	Black and White American	Total: 15,242 Black Americans: 3986, White Americans: 11,256	Total: 1827 (12.0%) Black Americans: 805 (20.2%). Males 18.7%, Females 21.1%. White Americans: 1022 (9.1%) Males 10.2%, Females 8.1%.	ARIC study baseline data (1987–89) was used (USA)	Men and women aged 45–65 years	–	ABSI, BAI, BMI, WC, WHTR, WHR (adjusted for age, physical activity and FHD)	ADA criteria: FPG ≥ 126 mg/dL, non-FBG ≥ 200 mg/dL, self-report of diabetes diagnosis, or self-report of taking diabetes medications
**4**	**Meeks, 2015** [32]	Ghanian, African Surinamese, Dutch	Total: 6213 Ghanaian (1873), African Surinamese (2189) and Dutch (2151)	Total: 562 (9.0%) Ghanian: 224 (12.0%), African Surinamese: 261 (12.0%), Dutch: 77 (3.6%)	Data from a multi-ethnic HELIUS cohort (The Netherlands)	Age: 18–70 years. Men and women	–	BF%, WHR	WHO criteria (2006): FPG ≥ 7.0 mmol/L, or current use of medication prescribed to treat diabetes, or self-reported diabetes. ADA criteria (2011) are equal to WHO criteria but adds individuals with a HbA1c of 6.5% (48 mmol/mol)
**5**	**Aggarwal, 2022** [15]	White, Asian, Black, Mexican, Other Hispanic American	Total: 19,335 White: 6319 Asian: 258 Black: 4597 Mexican: 2884 Other Hispanic American: 2114	Prevalence, % White: 9.1 (8.3–10.0) Asian: 11.9 (10.6–13.1) Black: 14.3 (13.0–15.7) Mexican: 12.8 (11.1–14.4) Hispanic: 10.7 (9.2–12.2)	Data from NHANES, 2011 to 2018 (USA)	Nonpregnant US adults aged 18 to 70 years. Men and women	–	BMI (normal, overweight, obese weight)	UDM: HbA1c level of ≥6.5%
**6**	**Bennet, 2014** [35]	Iraq (Arab), Sweden (White)	Total: 2147 Iraq (Arabic population): 1394; Sweden: 753	Total: 206 (9.6%) Iraq: 162 (11.6%); Sweden: 44 (5.8%)	Data was collected between 2010-2012 (Sweden)	Men and women born in Iraq or Sweden aged 30–75 years. Mean age at T2D diagnosis: 47.6 (9.7) for Arab and 53.4 (11.9) for White participants	–	WC, FHD. Adjusted for age, sex, insulin sensitivity index (ISI), corrected insulin response.	FPG level of 7.0 mmol/L and/or by a 2-h plasma glucose level of 11.1 mmol/L. If only one glucose value was pathologic, the OGTT was repeated on another day within 2 weeks
**7**	**Cheong, 2014** [61]	Malay, Chinese, Indian, Other indigenous	Total: 32,703 Malay: 17,959; Chinese: 6636; Indians: 2717; Other indigenous: 3747. Others: 1644	Total: 3727 (11.4%)	Data from the Third National Health and Morbidity Survey In 2006 (Malaysia)	18 years and older men and women	18 years and older men and women. Median age was 41 years.	WC (optimal cut-off to predict T2D by gender). Analysis was adjusted for age.	FPG ³6.1 mmol/l or known diabetes
**8**	**Cheong, 2013** [62]	Malay, Chinese, Indians, Other indigenous, Others	Total: 32,703 Malay: 17,959; Chinese: 6636; Indians: 2717; Other indigenous: 3747; Others: 1644	Total: 3727 (11.4%)	Data from the Third National Health and Morbidity Survey In 2006 (Malaysia)	18 years and older men and women. Median age 41 years. 45,8% men	–	BMI ( < 23 kg/m^2^; 23.00-24.99 kg/m^2^; 25.00–27.49 kg/m^2^; 27.50–29.99 kg/m^2^; 30.00 kg/m^2^). Analysis was adjusted for age.	FPG ³6.1 mmol/l
**9**	**Cohen, 2009** [56]	White, African American	Total: 64,435 19,589 African American men, 6202 White men, 27,021 African American women, and 11,623 White women	Total: 13,301 (20.6%) White man: 1159 (18.7%); African American men: 3302 (16.9%); White women: 2431 (20.9%); African American women: 6409 (23.7%)	Data Southern Community Cohort Study in from 2002 to 2009 (USA)	Aged 40 years and older men and women	–	Weight gain. Analysis was adjusted to age, BMI at 21years, education, income, smoking, physical activity level, hypertension, marital status.	Self-reported T2D
**10**	**Diaz, 2007** [59]	US white, US black, Mexican American, English white, English black, Indian, Pakistani, Bangladeshi, Chinese	Total: 11,624 US White: 710, US Black: 195, Mexican American: 184, English White: 2116, English Black: 134, Indian: 232, Pakistani: 88, Bangladeshi: 56, Chinese: 121.	Total: 3836 (33.0%) UDM: US White 2.7%, US Black 5.8%, Mexican American 6.2%, English White 2.3%, English Black 4.3%, Indian 4.1%, Pakistani 6.4%, Bangladeshi 5.9%, Chinese 1.5%	Data from 2003–2004 NHANES and 2003–2004 Health Survey (USA and UK)	20 years or older adults. Men and women.	–	BMI, WC, WHR. Unadjusted	Self-report of doctor diagnosis or HbA1c > 6.1%
**11**	**Gong, 2015** [47]	Uyghur, Han Chinese	Total: 5923 Uyghur: 2863, Han: 3060	Prevalence of T2D: Uyghurs 10.47%, Han 7.36%	Study was conducted in June-August 2013 (China). Response rate 88.6%	Adults aged 20–80 years. Men and women	–	BMI (adjusted for residency, age, TG, HDL-C, smoking status, drinking status, hypertension)	ADA 2009 criteria: FPG ≥ 7.0 mm/L (126 mg/dL), or/and a previous diagnosis of diabetes
**12**	**Hamoudi, 2019** [40]	UAE, Asian, Arab non-nationals	Total: 3203 Arab non-nationals (640), Asian (1683) and UAE nationals (797)	Total: 614 (19.2%) Arab non-national: 99 (15.5%). Asian: 272 (16.2%). UAE nationals: 243 (30.5%). Prevalence of newly diagnosed T2D was: UAE men 9.36%, UAE women 11.05%; Asian men 12.04%, Asian women 5.49%; Arab non-nationals men 13.46%, Arab non-nationals women 9.94%.	Data from UAE National Diabetes and Lifestyle Study (UAEDIAB), UAE	Phase one: all non-UAE national adults aged 18 years and older residing at least 4 years in the UAE. Phase two: 18 years or older UAE residents. Men and women	–	BMI, WC, FHD. Adjusted for age, sex, physical activity level, snoring, HDL, TG, high cholesterol, hypertension, SBP, DBP.	FBG ³7.0 mmol/l, ³6.5% HbA1c and participants’ self-report information
**13**	**Huxley, 2008** [63]	Asians and Whites	Total: 263,000	–	Data from Obesity in Asia Collaboration 21 study populations from 11 countries in the Asia-Pacific region (Iran, India, China, Thailand, Singapore, Australia, South Korea, Japan, Taiwan, Philippines)	Men and women	–	BMI, WC, WHR. Analysis adjusted to age.	FPG > 7 mmol/l. Individuals with the history of T2D or on diabetic medication were excluded
**14**	**Jenum, 2005** [34]	Western, South Asians	Total: 2513 Western: 2302, South Asians: 211	Western women: 2.9 (1.9–3.9), Western men 5.9 (4.2–7.5). South Asian women 27.5 (18.1–36.9), South Asian men 14.3 (8.0–20.7)	Data was collected via collected data using questionnaires, physical examinations and serum analyses (attendance rate 49.3%) (Norway)	30- to 67-year-old men and women in an area of Oslo with low SES	–	BMI, WHR, body height (adjusted for age, physical activity, SE factors (income, years of education))	Known diabetes: self-report. UDM: no previous diabetes and FSG of ³7.0 mmol/l, or HbA1c > 6.4%, or NFSG ³11.1 mmol/l and not attending for fasting venous samples
**15**	**Jenum, 2012** [46]	Norway, Turkey, Vietnam, Sri Lanka, Pakistan	Total: 4110 Norway: 1871, Turkey: 387, Vietnam: 553, Sri Lanka: 879, Pakistan: 420	Total: 406 (9.8%) (Prevalence was higher among Pakistan and Sri Lanka individuals, followed by Turkey, Vietnam and Norway subjects)	Data from the Romsås in Motion Study, Oslo and The Oslo Immigrant Health Study (subjects from Sri Lanka, Pakistan, Vietnam, Turkey and Iran living in Oslo, Norway)	Participants in the Romsås in Motion Study were 30–67 years old and in The Oslo Immigrant Health Study 30–60 years old men and women. Among known diabetes cases (238) 18 (6.8%) were diagnosed before the age of 25.	–	BMI, WHR x 10, WC, body height, waist-to-stature ratio (WSR) (known as WHTR), parity (0–2, 3, >4). Adjusted for age (cont.), part/full time work (yes/no), education ( > 9/9 years), heavy PA (yes/no).	Previous T2D: self-reported diabetes. UDM: FPG or HbA1c or with elevated non-FSG.
**16**	**Li, 2017** [48]	Non-Hispanic white, Non-Hispanic Asian, Others, Hispanic	Total: 1866 (1889) Non-Hispanic white: 498, Non-Hispanic Asian: 315, Others: 834, Hispanic: 219	The prevalence of diabetes in the Medicaid population was 10.3%, compared with 8.9% in the non-Medicaid population	Data: 2013 through 2015 from the Hawaii Behavioural Risk Factor Surveillance System (USA)	Adults aged 18 years or older. Men and women	–	BMI (adjusted for health insurance status, sex, age, marital status, check-up status, physical activity, immunisation, alcohol consumption)	Self-reported diabetes
**17**	**Lorenzo, 2007** [60]	Mexican American, non-Hispanic white	Total: 7233 SAHS: 2839 (Mexican-Americans and non-Hispanic whites); MCDS: 2233 (white subjects); SIRS: 2161 (Spain)	Among men: Mexico city: 12.8%, San Antonio Mexican Americans 13.7%, San Antonio non-Hispanic whites: 6.5%, Spain: 8.8%. Among women: Mexico city: 14.2%, San Antonio Mexican Americans: 17.0%, San-Antonio non-Hispanic whites: 5.3%, Spain: 6.7%	Data from 3 population-based studies: San Antonio Heart Study (SAHS) (USA), Mexico City Diabetes Study (MCDS) (Mexico) and Spanish Insulin Resistance Study (SIRS) (Spain)	SAHS: Mexican American and Non-Hispanic white men and non-pregnant women aged 25–64 years (response rate 65.3%). MCDS: 35–64 aged men and non-pregnant women (response rate 68.5%). SIRS: men and non-pregnant women of white ethnicity aged 35–64 years (response rate, 66.9%)	–	BMI, WC, WHR, WHTR	The 2003 ADA FPG criteria were used to diagnose DM (FPG ³7.0 mM/L, 2-hour glucose 11.1 mM/L) Or (1) treatment not with insulin but with oral anti-diabetic agents; (2) treatment with insulin plus age of diabetes onset 40 years or BMI 30 kg/m2 at enrolment
**18**	**Ntuk, 2017** [5]	Black, South Asian, White	Total: 418,656 Black: 7266 South Asian: 8540 White: 408,530	Total: 18,711 (4.5%). Black (total, % for men and women): 754, 11.8% and 9.4%. South Asian (total, % for men and women): 1282, 17.4% and 12.8%. White (total, % for men and women): 16,675, 5.4% and 3.0%.	UK Biobank (UK)	Men and women aged 40–69 years, who had complete data on diabetes status and hand-grip strength. Ethnicity was based on self-classification into the 19 UK Office of National Statistics groups.	–	Hand grip strength (absolute and relative estimates). Low grip strength was defined as grip strength below the age- and sex-specific overall UK Biobank population median (adjusted for age, education, number of years with diabetes, socio-economic status, percentage body fat, smoking, dietary intake, sleep duration and physical activity)	Self-report of a physician diagnosed T2D
**19**	**Ntuk, 2014** [4]	Black, South Asian, White	Total: 490,288 White: 471,174 South Asian: 9631 Black: 7949 Chinese: 1534	Total: 25,564 (5.2%) White: 22,880 (4.8%), South Asian: 1,686 (17.5%), Black: 906 (11.4%) Chinese: 92 (6.0%)	UK Biobank (UK)	40–69 years men and women	–	BMI, WC, percentage body fat, and WHR (adjusted for age, physical activity, SES, and heart disease)	Self-report of a physician diagnosed T2D
**20**	**Okosun, 1998** [76]	Jamaican, US/African Americans	Total: 3113 Jamaican: 1286 US/African Americans: 1827	-	Data from Ibadan, Nigeria, and local surveys in Spanish Town, Jamaica, and the U.S. NHANES III 1989, 1995 were used in this study. The Nigerian and Jamaican data were part of the International Collaborative Study of Hypert ension in Blacks (ICSHIB) 1994–1996.	25–74 aged men and women	–	WC (first quintile as a reference) (adjusted for age)	ADA criteria: (1) current diagnosis and use of insulin or hypoglycaemic agent or (2) FBG 126 mg/dl or a 2-h postload value of 200 mg/dl in the oral glucose tolerance test
**21**	**Ryckman, 2014** [66]	White, Black, Asian or Pacific Islander, Hispanic, Other/Unknown	Total: 75,993 White: 64,709, Black: 5579, Asian or Pacific Islander: 1840, Hispanic: 2586, Other/Unknown: 1279	Total: 4002 (5.3%) White: 2720 (4.2%), Black: 778 (14.0%), Asian or Pacific Islander: 141 (7.7%), Hispanic: 252 (9.7%), Other/Unknown: 111 (8.7%)	Data from the Women’s Health Initiative Observational Study (USA)	Postmenopausal women between the ages of 50 and 79.	–	Self-reported birth weight category (unknown, less than 6 pounds (lbs.), 6–7 lbs. 15 ounces (oz.), 8–9 lbs. 15 oz., and 10 or more lbs) (adjusted for age)	Self-reported physician diagnosed diabetes
**22**	**Strings, 2023** [77]	White, Black, Latin (combining the Mexican American and Other Hispanic groups)	Total: 45,514	–	Data from 10 waves of the continuous National Health and Nutrition Examination Survey from 1999-2000 through 2017–2018 (USA)	Men and women aged 18 years or older	–	BMI (adjusted for age, gender, and year of survey). Ethnicity was self-reported.	HbA1c ≥ 6.5%
**23**	**Vicks, 2022** [44]	Non-Hispanic White, Chinese, Filipino, South Asian	Total: 373,098 283,110 (non-Hispanic) White, 33,263 Chinese, 38,766 Filipino, and 17,959 South Asian	Total: 52,548 (14%) Among men. White: 18,870 (14.3%), Chinese:2,480 (17.5%), Filipino: 5370 (33.7%), South Asian: 2944 (31.7%). Among women. White: 13,426 (8.9%), Chinese: 1954 (10.2%), Filipino: 5717 (25.1%), South Asians 1787 (20.6%).	Data from integrated healthcare delivery system (EHR) Kaiser Permanente Northern California (KPNC), USA	Men and women aged 45–64 years who were members of a Northern California health plan in 2016	–	BMI (healthy weight, overweight, and obesity categories). BMI thresholds for White adults (18.5 to<25, 25 to <30, ≥30 kg/m2) and lower BMI thresholds for Asian adults (18.5 to <23, 23 to <27.5, ≥27.5 kg/m2). Underweight range was not included in the analysis (adjusted for age and BMI)	T2D was classified based on laboratory data, clinical diagnoses, or diabetes pharmacotherapy
**24**	**Yoon, 2016** [54]	Korean, White, Black, Hispanic	Total: 23,502 Korean: 18,845 White: 2347 Black: 854 Hispanic: 1456		Data from The 2007 —2010 United States NHANES, USA, and the 2007—2010 Korea National Health and Nutrition Examination Survey (KNHANES), Korea	Adults 20 years or older. Men and women	–	BMI, WC. (adjusted for age, sex, alcohol intake, current smoking status, physical activity, sleep duration, education level, medication of antihypertensive drug, medication for dyslipidaemia, and total daily energy intake, HOMA-IR, HOMA-b)	FPG ≥ 126 mg/dL
**25**	**Zethof, 2021** [39]	African Surinamese, South Asian Surinamese, Turkish, Moroccan, Ghanaian, and Dutch origin. Ethnicity was self-reported.	Total: 21,072 African Surinamese: 3997, South Asian Surinamese: 2956, Turkish:3546, Moroccan: 3850, Ghanaian: 2271, Dutch: 4452.	Total prevalence was 22.1%. Among men. Dutch 5.5%, South Asian Surinamese 23.6%, African Surinamese 14%, Ghanian 17.8%, Turkish 12.3%, Moroccan 13.2%. Among women. Dutch 14.2%, South Asian Surinamese 20.9%, African Surinamese 14.4%, Ghanian 12.4%, Turkish 10.5%, Moroccan 11.8%.	Participants from HELIUS, the Netherlands	Men and women aged between 18 and 70 years. Age at T2D diagnosis among men: Dutch 44.26, South Asian Surinamese 43.76, African Surinamese 44.77, Ghanian 45.05, Turkish 41.96, Moroccan 41,87. Age at T2D diagnosis among women: Dutch 49,39, South Asian Surinamese 43.2, African Surinamese 45.03, Ghanian 44.34, Turkish 42.47, Moroccan 41,17	–	WHR, WC, BMI, body fat % (adjusted for age)	WHO criteria: FPG level ³7.0 mmol/L, had self-reported diabetes, or use of glucose-lowering medication
**26**	**Signorello, 2007** [49]	Whites, African Americans	Total: 43,822 White men: 3165 African Americans men: 14,236 White women: 6326 African American women: 20,095	Total: 9223 (21%) White men: 20% African American men: 17% White women: 21% African American women: 24%	Data from Southern Community Cohort Study (2002–2006), USA	Men and women 40 and 79 years old (mean age 51.2 years (SD = 8.7))	–	BMI (adjusted for age, educational level, household income, Nam–Powers–Boyd occupational status score, health insurance coverage, body mass index at age 21, hypertension, time per week engaged in moderate sports in 30 s, and time per week engaged in vigorous sports in 30 s)	Self-reported physician diagnosed diabetes
**27**	**Steinbrecher, 2015** [55]	Whites, Native Hawaiians, Japanese Americans	Total: 40,455 White: 16,314 (40.3%), Native Hawaiian: 4970 (12.3%), Japanese American: 19,171 (47.4%)	Total: 4651 (11.5%)	Data from the Multi-ethnic Cohort Study (Hawaii component), USA. Response rates ranged from 28 to 51% in the different ethnic-sex groups	Men and women aged 45–75 years	–	BMI, WC, HC, WHR, WHTR. (adjusted for age, ethnicity, education, physical activity, hypertension, processed red meat intake, dietary fibre intake, smoking and alcohol intake)	Self-reports and by linkages with health insurance plans
**28**	**Nyamdorj, 2010** [45]	Asian Indian, Chinese, Mauritian Indian, European, Japanese	Total: 54,467 (24 515 men and 29 952 women) Chinese: 4514 Japanese: 3001 Mauritian Indian: 2123 Asian Indian: 5085 European: 9792	Crude prevalence (total) of undiagnosed diabetes ranged from 9.5 to 13.2% among the Asian Indians living in India and Mauritius, from 4.7 to 10.2% in the Japanese and Chinese, and from 4.7 to 6.4% in the Europeans	Data from the population-based DECODA and the DECODE studies (International)	Men and women aged ³30 years	–	BMI, WC (adjusted to age and stratified by gender)	UDM: FPG concentration of ³7.00 mmol l-1 and/or a 2-h post- load plasma glucose concentration of ³11.10 mmol l-1 following a 75-g OGTT.
**29**	**Zhu, 2019** [50]	White, Black, Hispanic, Asian, Hawaiian/Pacific Islander, American Indian/Alaskan Native	Total: 4,906,238 White: 2,454,388; Black:467,994; Hispanic: 1,058,351; Asian: 620,813; Hawaiian/Pacific islander: 67,190; AI/AN: 26,324	Total: 15.9% (age-standardized) White: 12.2%, Black 21.4%, Hispanic 22.2%, Asian 19.3%, Hawaiian/Pacific Islander: 27.7%, AI/AN: 19.6%	Data from the Patient Outcomes Research To Advance Learning (PORTAL) Network, one of the 13 Clinical Data Research Networks in the National Patient-Centred Clinical Research Network, USA	Men and women aged ≥20 years	–	BMI categories (underweight, normal, overweight, obese class 1, obese class 2, obese class 3, obese class 4) (adjusted for age, sex, neighbourhood poverty, neighbourhood education, and site)	ICD-9 codes or any combination of two other events: fasting plasma glucose $126 mg/dL ($7.0 mmol/L), random plasma glucose $200 mg/dL ($11.1 mmol/L), HbA1c $6.5%, outpatient diagnosis (the same as in- patient codes), or dispensation of an antihyperglycemic medication
**Case-control studies**									
**30**	**Abdullah, 2018** [78]	Malay, Chinese, Indian	Total: 4077 (1962 cases and 2115 controls) Malay: 1323 Chinese: 1344 Indian: 1410	Total: 1962 Malay: 600 Chinese: 654 Indian: 708	Participants from the Malaysian Cohort project, a prospective multi-ethnic, population-based cohort (Malaysia)	35–70 years old adults (men and women). For all ethnic groups the highest proportion of cases were diagnosed between 50-60 years	–	WC, WHR, FHD. Adjusted for age, gender, physical activity, sleep duration and location of study.	FPG ≥ 7.5 mmol/L (or 126 mg/dL); a similar number of ancestries matched controls: FPG < 5.5 mmol/L (or 99 mg/dL) without previously diagnosed diabetes
**31**	**Mayer-Davis, 2008** [65]	African American, Hispanic, non-Hispanic white	Total: 247 Cases: 80 Controls: 167 (African American: 28.7%, Hispanic: 18.6%, Non-Hispanic white: 50.9%)	Total: 80 African American: 51.3%, Hispanic: 20.0%, Non-Hispanic white: 28.8%	Multicentre SEARCH study was conducted in 2001 (Columbia, USA)	Youths aged 10-21 years. Men and women	–	Breast-feeding (yes/no) status and its duration (Reported by biological mother)	Type of diabetes was based on provider diagnosis. Using Health Insurance Portability and Accountability Act– compliant procedures, youth with diabetes identified by the SEARCH recruiting network
**32**	**Marshal, 1993** [79]	Non-Hispanic white, Hispanic	Total: 767 (cases: 279 controls: 488) Non-Hispanic whites: 391 Hispanics: 376	Total 279 Non-Hispanic Whites: 92 Hispanics: 187	The San Luis Valley Diabetes Study, USA, 1984-1986	20–74 aged men and women. Controls were selected via two-stage sampling and randomly selected to reflect the age structure of cases.	–	BMI (5-unit increase), subscapular skinfold (5-mm increase), triceps skinfold (5-mm increase), subscapular/triceps skinfold ratio (0.2-unit increase), WHR (0.1-unit increase), FHD (adjusted for sex, education, annual income)	Diabetes was defined via health records or self-report
**33**	**Paul, 2017** [43]	White European, African Caribbean, South Asian	Total: 452,915 (90,367/362,548) White European: 396,350 African-Caribbean: 20,575 South Asian: 36,260	Total: 90 367 White European: 79,270 African-Caribbean: 4115 South Asian: 7252	Data was obtained from the Health Improvement Network (THIN) database, a large anonymized longitudinal dataset (UK)	18 years or older UK residents. 56% were males, 44% females. Mean age at diagnosis was 58, 48 and 46 years for whites, African American and South Asians, respectively.	–	BMI at the time of diagnosis of T2D (adjusted for sex, smoking status, deprivation score and history of CVD, cancer and CKD on or prior to the index date)	T2D was diagnosed with the following algorithm: patients with Read Codes related to T2D, and if they received at least 1 prescription of antidiabetic medications or received a lifestyle modification intervention
**Cohort studies**									
**34**	**Caleyachetty, 2021** [9]	White, South Asian, Black, Chinese, Arab	Total: 1,472,819. White: 1333 816 (90,6%), South Asians: 75 956 (5,2%), Black: 49 349 (3,4%), Chinese: 10 934 (0,7%), Arab: 2764 (0,2%)	Total: 97,823 (6.6%). White 89,287 (6.7%), South Asians 5632 (7.4%), Black 2444 (5.0%), Chinese 317 (2.9%), Arab 143 (5.2%).	Data from EHR CPRD linked to hospital episode statistics, UK	Men and women aged 18 years or older, without past/current diagnosis of T2D and BMI between 15 and 50 kg/m². The median age at diagnosis for Whites 67 years (IQR 57–76), South Asians 55 years [45–65], Black 54 years [47–65], Chinese 60 years [52–68], and Arab 56 years [47–64]	Median follow-up 6,5 years (IQR 3,2–11,2)	BMI (adjusted for age and sex)	T2D cases were identified by use of a CALIBRE phenotyping algorithm (a combination of a general practitioner diagnosis of T2D and ICD-10 codes)
**35**	**Dreyfus, 2012** [30]	African American, White	Total: 8491 African American: 2505 White: 5986	Total: 990 (11.6%) African American: 508 (20.3%) White: 482 (8.1%)	Participants were selected from the ARIC cohort study (USA)	Women with mean age 54 years. Excluded: women with missing age at menarche (*n* = 49), or who reported their age at menarche as 18 years (*n* = 11), those with diabetes diagnosed before the age of 30 years (*n* = 30) (potential T1D). Mean age at diagnosis for African Americans was 49.8 [9], for Whites – 51.3 [9]	9-years of follow-up	Age at menarche (adjusted to age and study centre, BMI)	Self-reported history of physician-diagnosed diabetes, FPG ≥ 7.0 mmol/l (126 mg/dl), non-FPG > 11.1 mmol/l (200 mg/dl) or self-reported use of hypoglycaemic medication in the 2 weeks before the visit for a maximum of 9 years of follow-up
**36**	**Hardy, 2017** [51]	Black Americans, White Americans	Total: 12,121 Black Americans: 2630 White Americans: 9491	Total: 1359 (11.2%) Black Americans: 427 or 16.2% (Males 15.39%, Females 16.77%) White Americans: 932 or 9.8% (Males 11.73%, Females 8.13%)	ARIC study baseline data was used (USA)	Men and women aged 45–65 years without diabetes at baseline. Mean age was 54 (SD 5.7) years	11 years of follow-up	ABSI, BAI, BMI, WC, WHTR, WHR, WHHR (adjusted for age)	ADA criteria: FPG ≥ 126 mg/dL, non-FBG ≥ 200 mg/dL, self-report of diabetes diagnosis, or self-report of taking diabetes medications
**37**	**MacKay, 2010** [42]	Non-Hispanic White, African American, Hispanic	Total: 1,073 Non-Hispanic white: 430, African American: 282, Hispanic: 361	Total: 146 (13.6%)	The Insulin Resistance Atherosclerosis Study (IRAS), USA	Nondiabetic subjects aged 40–69 years at baseline (1992– 1994). Men and women	5,2 years	WHTR, BMI, WC, WHR, HC	1999 WHO criteria for 2 h OGTT
**38**	**Almahmeed, 2017** [64]	South Asians, Chinese, White	Total: 738,440 Chinese: 34,474; South Asian: 29,474; White: 674,492	DM incidence: Chinese: 1.9%, South Asian: 3.8%, White: 1.9%	Along with primary collected data, Inpatient and outpatient medical records were used (Canada)	Women aged 18–50 years	Follow-up 10 years (from 180 days after index delivery and censored at the first occurrence of the following events: death; loss of insurance coverage or having reached the follow-up)	The index pregnancy (1, 2, 3, 4 and ≥5 deliveries). Analysis was adjusted to age, income, rural residency, recency of immigration, GDM, pre-existing (chronic) hypertension, gestational hypertension, other comorbidities and Healthcare utilization	The diagnosis of diabetes was made if there was one hospitalization or two outpatient physician service records within 2 years bearing a diagnosis of T2D
**39**	**Chan, 2018** [28]	Malay, Indian	Total: 4101 (Malay 1901 and Indian 2200)	Total: 308 (cum.inc. 12.8%) Malays: 132 (10.9%) Indian: 176 (14.7%)	Data from two propective cohort studies: SiMES, 2004–2006, SiME-2, 2011–2013, and the SINDI, 2007–2009, SINDI-2, 2012– 2015 (Singapore)	Mean age of T2D patients was 54.5 years, without diabetes – 55.1 years. Men and women.	Median 6.2 years of follow-up	BMI (underweight/normal, overweight, obese weight). Adjusted for age, gender, ethnicity, family history of diabetes, income, education, current smoking status, systolic blood pressure, HbA1c, total cholesterol, HDL cholesterol and diabetes duration	Random plasma glucose ≥200 mg/dL, HbA1c ≥ 6.5% or self-reported physician diagnosed DM.
**40**	**Chiu, 2011** [29]	White, South Asian, Chinese, Black	Total: 59,824 White: 57,210 South Asian: 1001 Chinese: 866 Black: 747	Total: 4,076 (cum.inc. 6.8%) Incidence of T2D: White 9.5 (9.1–9.9), South Asian 20.8 (16.1–25.4), Chinese 9.3 (5.8–13.1), Black 16.3 (11.8–21.6)	Participants from Statistics Canada’s 1996 National Population Health Survey (NPHS) and the Canadian Community Health Survey cycles (2001, 2003, 2005), Canada. Survey data was linked to EHRs	Men and women without baseline diabetes aged 30 years or older. Median age at diagnosis: 49 years among South Asians, 55 years among Chinese, 57 years among Black and 58 years among Whites	12.8 years. Median follow-up 6 years.	Identification of optimal BMI cut-off values	ICD codes from EHRs
**41**	**Kulick, 2016** [80]	Non-Hispanic whites, black non-Hispanics, Hispanics	Total: 2.430 Non-Hispanic whites: 563, Black non-Hispanic: 576, Hispanic: 1291	Total: 449 (cum.inc. 18.5%) 43 (7.6%) diagnoses of diabetes in non-Hispanic whites, 77 (13.4%) in non-Hispanic blacks, and 329 (25.5%) in Hispanics	Participants in the Northern Manhattan Study without diabetes at baseline was studied from 1993-2014 (USA). Overall enrolment rate was 68%	Individuals who never had stroke and were at least 40 years old. Ethnicity was self-reported. Mean age 69 years. 37% were men	11 years	BMI (sociodemographic, CVD risk factors, CRP)	Self-reported diabetes
**42**	**Luo, 2019** [52]	NHW, American Indian/Alaska Native, Asian, Black or African American, Hispanic/Latina,	Total: 136,112 NHW: 115,412; AI/AN: 524; Asian: 3484; Black or African American: 11,370; Hispanic/Latina: 5322	Total: 18,706 Black women 1.7%, AI/AN 1.5%	Participants were drawn from Women’s Health Initiative prospective cohort study (1993–1998), USA	50–79 years old postmenopausal women. Race/ethnicity was self-reported.	14.6 years	BMI, WC, WHR, whole-body fat, whole body fat percent, trunk fat, trunk-to-leg fat ratio (adjusted for age at enrolment, education level, FHD, different study cohorts, smoking, alcohol intake, physical activity, HEI-2005 score, high cholesterol level, medicines use)	Self-reported diabetes
**43**	**Lutsey, 2010** [53]	White, Chinese, Black, Hispanic	Total: 546 Analysis was shown for 5603. Whites: 2360; Chinese: 646; Black: 1442; Hispanic: 1155	Total: 479 (cum.inc. 8.5%) (147 (6.2%) in whites, 150 (10.4%) in blacks, 48 (7.4%) in Chinese, and 134 (11.6%) in Hispanics)	Data from the MESA initiated in 2000, USA	45-84 years US men and women	6.6 years (median 4.7)	BMI, WC (adjusted for age, sex, race/ethnicity, education, and income)	Incident diabetes: Participants taking diabetes medications or whose glucose, after a minimum 8-hour fast, was ³126 mg/dL at any of the follow-up examinations
**44**	**Ma, 2012** [23]	White, Black, Hispanic, Asian	Total: 158,833 White (NHW): 133,541 Black (NHB): 14,618 Hispanic: 6484. Asian: 4190	Total 14,604 (cum.inc.9.2%) White: 11,127 (8.63%), Hispanic: 879 (14.63%) Black: 2181 (17.01%) Asian: 417 (10.58%)	Participants from the Women’s Health Initiative Study (USA)	50–79 years old postmenopausal women. The average age was 63 at baseline	Average 10.4 years	BMI ( < 25 kg/m2 vs. ³25 kg/m2)	Self-reported diabetes
**45**	**Maskarinec, 2009** [67]	White, Japanese American, Native American, Hawaiian, Others	Total: 103, 898 White: 35,042 Japanese American: 44,513 Native Hawaiian: 14,346 Other: 9997	Total: 11,838 (cum.inc.11.4%) White: 2386 (5.8 [5.0–6.6]), Japanese American: 5957 (12.5 [11.4–13.5]), Native Hawaiian: 2182 (15.5 [13.3–17.6]), Other: 1313 (12.2 [9.9-14.4])	Participants from Hawaii component of the Multi-ethnic Cohort, (1993-1996 followed until 2007), USA	47% men and 53% women	Mean follow-up 11.9 ± 3.4	BMI (adjusted for age, sex, and education)	Self-report and the information obtained through the health plan linkages
**46**	**Morimoto, 2011** [57]	Native Hawaiians, Whites, Japanese Americans	Total: 78,006 Native Hawaiians: 10,877; Whites: 30,715; Japanese Americans: 36,414	Total: 8892 (11.4%) Native Hawaiians: 1,792 (16.5%) Whites: 1870 (6.1%) Japanese Americans: 5230 (14.4%) By gender (men/women, in %): White 7.2/5.0, Japanese American 16.1/12.8, Native Hawaiian 17.2/15.9.	The Multi-ethnic Cohort Study, 1993-1996 (with 5.5 years of follow-up), Hawaii (USA)	45–75 years individuals. Men and women	5.5 years ( ± 0.8)	Self-reported weight gain (at baseline of the study, at age 21 and after 5.5 years) (adjusted for gender, age, education, physical activity and BMI at age 21)	Diabetes was diagnosed through self-report and a linkage to medical databases
**47**	**Narayan, 2021** [81]	South Asian, White, Black	Total: 16,119 South Asian: 3.136 White: 9924 Black: 3.059	Total: 2,013 South Asians (total, age-adjustedd incidence for men/women): 389 (26 [22.2–29.8] and 31.9 [27.5–36.2]) White (total, age-adjusted incidence for men/women): 1036 (16.1 [14.8–17.4] and 11.3 [10.2–12.3]) Black (total, age-adjusted incidence for men/women): 588 (26.2 [22.7–29.7] and 28.6 [25.7–31.6])	Prospective Centre for Cardiometabolic Risk Reduction in South Asia Study (CARRS) and the ARIC Study (India, Pakistan and US)	Men and women free of diabetes at baseline and aged 45 years	4.8 (3.8–5.1) years in CARRS and median follow-up time: 8.8 years, IQR 5.6–9.0 years in ARIC study	BMI, FHD (adjusted for age, sex, log-HOMA-IR, log-HOMA-B)	In the CARRS and ARIC cohorts, incident diabetes was defined as FPG ≥ 7.00 mmol/L (126 mg/dL), HbA1c ≥ 6.5% (48 mmol/mol) or self-reported diabetes or on medication during follow-up
**48**	**Resnick, 1998** [33]	Black, White	Total: 11,383 1531 black and 9852 white subjects	Total: 1139 (10%) Black: 260 (17%), White: 879 (9%)	National Health and Nutrition Examination Survey, Epidemiologic Follow-up Study (1971–1992), USA	Men and women who were ages 25–74 years during the NHANES I and who completed the baseline medical examination	20 years of follow-up	BMI and subscapular-to-triceps skinfold ratio (STR) (adjusted for age)	Self-report, health record data or death certificates
**49**	**Rodriguez, 2021** [82]	White, African American, Hispanic, Chinese Americans	Total: 5659 White: 2383 African American: 1462 Hispanic: 1161 Chinese Americans: 653	Total: 696 (12.3%)	MESA cohort (2000–2001), USA	45–84 years men and women. Mean age was 62 years	42 686 person-years/11 years of follow-up	BMI. Ethnicity was self-reported.	FPG ≥ 7.0 mmol/l, or use of any diabetes medications
**50**	**Shaten, 1993** [83]	Blacks, non-Blacks	Total: 6000 White: 5420; Black: 428 Asian: 56 Hispanic: 70 Other: 26	Total: 4.1% or 247 cases Blacks: 28 or 6.5%. Non-Blacks (all other ethnicities combined): 219 or 3.9%	Data/participants from the Usual Care group of the Multiple Risk Intervention Trial (USA)	Men only. Average age was 46 years, BMI 27.6 kg/m^2^	5 years	Self-reported parental history of diabetes	T2D was defined as use of insulin/hypoglycemic agents, FPG ³140 mg/dl on two consecutive annual visits, or FPG ³140 mg/dl followed the next year by insulin or hypoglycaemic use
**51**	**Stevens, 2008** [38]	Chinese Asians, American Whites, American Blacks	Total: 20,338 Chinese Asians: 5980, American Whites: 10,776, American Blacks: 3582	Cumulative incidence: Chinese Asians 5.8 (4.5–7.1), American Whites 4.3 (3.7–5.0), American Blacks 8.7 (7.0–10.3)	People’s Republic of China Study (1983–1994), China, and the Atherosclerosis Risk in Communities Study (1987–1998), USA	Men and women aged 45–64 years at baseline	Average 8 years of follow-up	BMI (adjusted for gender, baseline age, education, smoking status, alcohol consumption, field centre)	FPG ³126 mg/dl, reported taking diabetes medication, or self-reported physician diagnosed diabetes
**52**	**Tillin, 2014** [41]	Europeans, South Asians, African-Caribbeans	Total: 2533 (1356 Europeans, 842 South Asians, 335 African-Caribbeans)	Diabetes incidence rates (per 1000 person years) were 20.8 (95% CI: 18.4, 23.6) and 12.0 (8.3, 17.2) in South Asian men and women, 16.5 (12.7, 21.4) and 17.5 (13.0, 23.7) in African-Caribbean men and women, and 7.4 (6.3, 8.7), and 7.2 (5.3, 9.8) in European men and women	Southall and Brent Revisited (SABRE) is a population-based cohort of Europeans, South Asians and African- Caribbeans from North and West London (UK)	Men and women aged 40-69 years at baseline (1988– 1991) All South Asians and African-Caribbeans were first-generation migrants. Ethnicity was confirmed based on parental origins	19 years of follow-up	BMI categories (using suggested WHO cut-points of <23, 25, 27.5 and 30 kg/m2) and by baseline WC in 10 cm categories (adjusted for sex and age)	Primary care records, participant recall and/or follow-up biochemistry
**53**	**Wei, 2015** [58]	Black, White	Total: 17,404 Black: 3655; White: 13,749	Diabetes incidence per 1000 PY in the younger and middle-aged groups was 7.2 (95% CI 5.7, 8.7) and 24.4 (22.0, 26.8) in blacks, respectively, and 3.4 (2.8,4.0) and 10.5 (9.9, 11.2) in whites, respectively	Participants from ARIC, CARDIA, and the Framingham Heart Study (USA)	56% women, 44% men The age ranges in ARIC, CARDIA, and the Framingham Heart Study were 44-59, 30-46, and 30-59 years, respectively	Median follow-up 9 years	Duration and degree of weight gain. Models were adjusted for sex, baseline HDL-C, log(triglycerides), fasting Ng glucose, prehypertension, hypertension, log (BMI-years below baseline BMI), as well as for interaction terms	FBG ³126 mg/dL, casual blood glucose ³200 mg/dL, or using insulin or oral hypoglycaemic medication
**54**	**Zamora-Kapoor, 2018** [84]	Non-Hispanic Whites, non-Hispanic blacks, AI/AN	Total: 8337 Non-Hispanic Whites: 5131 Non-Hispanic Blacks: 1651 Hispanics: 1223 American Indians/Alaska Natives: 332	Total: 484 Diabetes was more prevalent in non-Hispanic Blacks (12%) than in American Indians/Alaska Natives (11%), Hispanics (6%), and non-Hispanic Whites (3%)	the Add Health Study (1994–2008), USA	Males and females were 11–20 years old (mean age: 16 years) at baseline in 1994 and were followed for additional waves of data collection in 1996, 2002, and 2008. Retention rates in all waves was between 72% and 79%. 53% were females	Mean follow-up time was 14 years	BMI, FHD. Age, gender, physical activity level, parental education and financial instability. Both parents and adolescents completed questionnaires	HbA1C ≥ 6.5%, glucose > 125 mg/dl, self-reported diabetes, or self-reported diabetes medication use

*UDM* undiagnosed diabetes, *GDM* gestational diabetes, *BMI* body mass index, *WC* waist circumference, *WHR* waist-to-hip ratio, *WHTR* waist-to-height ratio, *WHHR* waist-to-hip-to-height ratio, *WHO* World Health Organisation, *IQR* interquartile range, *FPG* fasting plasma glucose, *OGTT* oral glucose tolerance test, *HC* hip circumference, *ABSI* a body shape index, *BAI* body adiposity index, *FHD* family history of diabetes, *ADA* American Diabetes Association, *TG* triglycerides, *HDL-C* high-density-lipoprotein cholesterol, *FBG* fasting blood glucose, *FSG* fasting serum glucose, *NFSG* non-fasting serum glucose, *SBP* systolic blood pressure, *DBP* diastolic blood pressure, *CRP* C-reactive protein, *SES* socio-economic status, *HER* electronic health records, *CVD* cardiovascular diseases, *CKD* chronic kidney diseases, *NHW* non-Hispanic white, *NHB* non-Hispanic black, *DREAM* Diabetes Reduction Assessment with Ramipril and Rosiglitazone Medication, *ARIC* Atherosclerosis Risk in Communities, *HELIUS* Healthy Life in an Urban Setting, *NHANES* National Health and Nutrition Examination Survey, *MESA* Multi-Ethnic Study of Atherosclerosis.

The results below focus on anthropometric measures such as BMI, waist circumference (WC), WHR, waist-to-height ratio (WHTR), weight gain, body fat percentage (BF%) (*n* = 50) and non-anthropometric factors related to women’s health (*n* = 5). Results from studies on other factors with less evidence (one study per risk factor) are reported in Table S3. The number of studies per anthropometric measure is shown in Table S4.

Among case-control studies, three had a moderate risk of bias and one had a low risk of bias. Biases related to high non-response rates differed between cases and controls. Among cohort studies, 12 had a low risk of bias and nine had a moderate risk of bias. Bias was mainly due to differences in the ascertainment of exposure, and the inability to provide evidence that an outcome of interest was not present at the beginning of the study. Among cross-sectional studies, risk of bias was high in seven studies, moderate in eight studies, and low in 14 studies The reasons for potential bias were the absence of standard criteria to measure the risk factor, or the design of the study did not consider and adjust for potential confounding factors. (Tables S5-7).

### Descriptive differences across ethnicities in incidence, prevalence, age at diagnosis and anthropometric measures

Prevalence and incidence rates of T2D varied across studies, ethnic groups, and sexes. T2D prevalence was two to four times higher in South Asian, Southeast Asian, Black, and other ethnicities compared to White individuals [9, 15, 29–40]. T2D incidence was higher in men among White populations but higher in women among Black populations, with mixed evidence for sex differences amongst South Asians [41, 42] (Table 1).

Median age of T2D onset was highest for people of White ethnicity, followed by East Asian, Black, Arab, Southeast Asian and South Asian ethnicities [9, 29, 37]. Similarly, the mean age of T2D onset was highest for White individuals (51–58 years), followed by Black (48–54 years), South Asian (44–46 years) and youngest among Turkish and Moroccan individuals (41–42 years) [30, 35, 36, 39, 43] (Table 1).

The age-standardized prevalence of T2D in each category of BMI and WC was two to threefold higher in non-white ethnic groups compared to White groups [15, 38, 41, 44–46]. Moreover, for any given age of diagnosis, mean BMI was lower for South Asian and Black ethnicities compared with White populations [43].

### Meta-analysis results

#### The relationship between BMI and T2D is strongest in white Europeans and varies across ethnicities

**Fig. 2:**
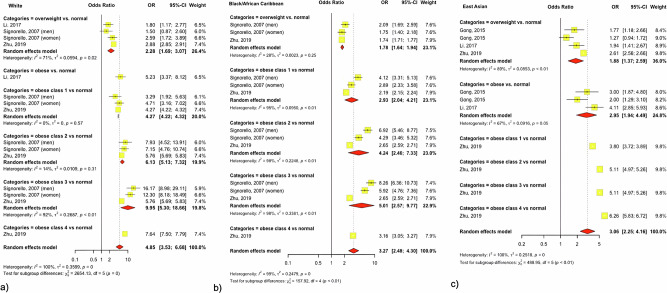
Trans-ethnic meta-analysis of the effect of overweight or obese vs. normal BMI on type 2 diabetes. **a** White, **b** Black and **c** East Asian ethnic groups).

Ethnicity-stratified meta-analysis for the association between BMI and T2D was conducted for people of White, Black and East Asian ethnicities (*n* = 4 studies). People of White ethnicity consistently had the highest odds of T2D within each BMI category compared to people of Black and East Asian ethnicities, however, confidence intervals overlapped suggesting limited evidence for ethnic differences in the association between BMI and T2D. When comparing people in obesity category 3 (BMI 40.0–49.9 kg/m^2^) to people with normal weight (BMI 18.5–24.9 kg/m^2^), odds of T2D were increased tenfold for people of White ethnicity (OR 9.95, 95% 5.30-18.66), and fivefold for other ethnic groups (Black OR 5.01,95% 2.57–9.77, East Asian OR 5.11, 95% 4.97–5.26) (Fig. 2) [47–50]. The included studies had high heterogeneity (I^2^ > 90%) and most of them a low risk of bias, suggesting the relationship between BMI and T2D could vary across ethnicities. Since only one study reported sex-stratified results, subgroup analyses by sex were not possible [49]. Meta-analysis results of studies reporting hazard ratios (HR, *n* = 2) and relative risks (RR, *n* = 2) per unit increase in BMI are shown in Supplementary materials (Fig. S1a-b). The funnel plots showed a possible publication bias (Fig. S2). Results from single studies not included in the meta-analysis are described in Tables S8–9 (*n* = 18 studies).

#### The association of WHR and T2D is stronger in people of Black compared to white ethnicity

**Fig. 3:**
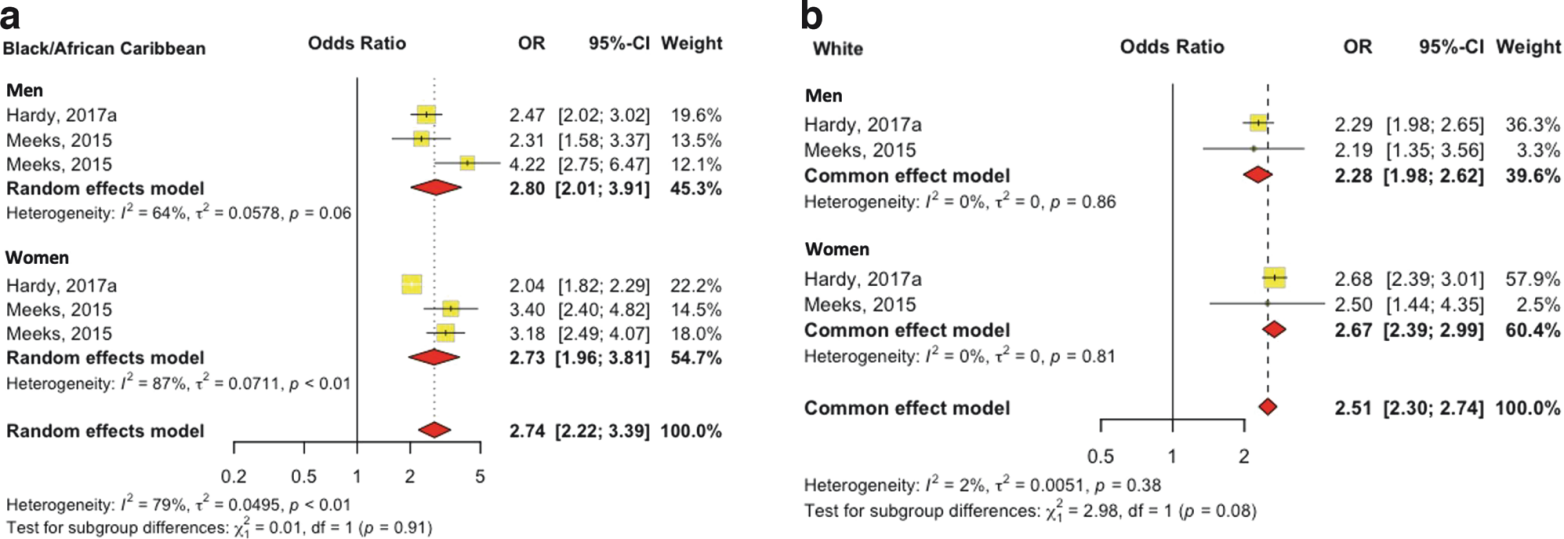
Trans-ethnic meta-analysis of the effect of WHR on type 2 diabetes. **a** Black ethnic group; **b** White ethnic group, per unit increase in WHR).

Two studies reporting overall and sex-stratified associations between WHR and T2D for people of White and Black ethnicity were meta-analysed [31, 32]. The odds of T2D associated with a one-unit increase in WHR were higher for people of Black ethnicity (OR 2.74, 95% CI 2.22–3.39) compared to people of White ethnicity (OR 2.51, 95% CI 2.30–2.74), with associations being strong among both sexes (Fig. 3). Studies for people of White ethnicity were homogeneous (I2 = 2%), while high heterogeneity was observed for studies including people of Black ethnicity (I2 = 79%). Most of the included studies had a low risk of bias and potentially publication bias (Fig. S2). Results from studies examining the association between WHR and T2D not included in the meta-analysis are described in Table S10 (*n* = 7 studies).

#### Narrative synthesis results

##### The effect of WC on T2D differs across ethnicities

Nine studies reported on ethnic differences in the association between WC and T2D, with potential interactions between WC and sex (Table S11) [31, 35, 40, 42, 45, 51–54]. Studies reported a stronger association between WC and T2D for East Asian, South Asian, and White individuals than for Hispanic, Native Hawaiian, and Black individuals [31, 35, 40, 42, 45, 51–54]. This trend was more pronounced among women [45, 55]. The Women’s Health Initiative study of postmenopausal women aged 50–79 years observed the strongest association between WC and T2D in East Asians and the weakest in Black women, with American Indian/Alaska Native and Hispanic participants having similar risk to White individuals [52]. Despite consistent trends, the overall quality of evidence is low to moderate due to study heterogeneity and bias.

##### WHTR is an independent risk factor for T2D across sexes and ethnic groups

Four studies reported on ethnic differences in the association between WHTR and T2D (Table S12). Findings from the Atherosclerosis Risk in Communities (ARIC) [31, 51], the Insulin Resistance Atherosclerosis Study (IRAS) [42] and Multi-Ethnic Cohort (MEC) studies [55] including individuals of White, Black, Hispanic, Japanese and Hawaiian ethnicities demonstrated that WHTR was an independent predictor of T2D across sexes and ethnic groups. In the ARIC [31, 51] and IRAS [42] cohorts, people of White ethnicity had higher odds of T2D associated with WHTR than individuals of Black ethnicity. Despite the low risk of bias in these studies, the evidence for WHTR’s predictive ability across ethnicities is insufficient due to the limited number of studies comparing the same ethnicities and the cross-sectional design of some.

##### The effect of body fat percentage on T2D was stronger among women than men and in White individuals compared to other ethnicities

Four studies reported on ethnic differences in the association between BF% and T2D (Table S13). All studies showed a stronger association for White individuals compared to all other ethnic groups, with the association stronger for women compared to men [32, 39, 42, 52]. The IRAS study found this association stronger in White than in Hispanic and Black individuals [42]. The HELIUS study reported a stronger association in White women compared to Black women, with no significant difference among men [32]. Similar trends were seen in a Dutch cross-sectional study [39] and the Women’s Health Initiative cohort [52], where T2D risk in Black women was weakly associated with BF% but strongly associated with trunk-to-leg ratio (*p* < 0.05 for interaction). The strength of association between T2D and BF % for White (HR 1.38, 95% CI 1.29–1.48) and Hispanic (HR 1.40, 95% CI 1.09–1.80) women was similar [52]. Despite consistent trends, the evidence quality was low to moderate due to cross-sectional designs and moderate/high risk of bias.

##### Weight gain is more strongly associated with T2D in white populations

Three studies reported on the association between weight gain and T2D, which was found to be stronger in Japanese and native Hawaiians, followed by individuals of White and Black ethnicities [56–58] Among people of White ethnicity, the association between weight gain and T2D was stronger among women than in men, while the opposite was observed among people of Black ethnicity: for 40+ kg weight gain compared to stable weight ( < 5 kg change) OR 4.0 (CI 3.2–4.9) vs. 3.6 (CI 2.7–4.8) and OR 2.6 (CI 2.3–3.1) vs. 3.3 (CI 2.8–4.0), respectively [56]. Similar results were reported in the study which combined ARIC, Coronary Artery Risk Development in Young Adults (CARDIA) and Framingham cohorts, with the association between weight gain and T2D stronger among White individuals than Black subjects: HR per one-unit increment in BMI-years were 1.18 (*p* = 0.02) for younger white individuals, 1.02 (*p* = 0.39) for middle-aged White individuals, 1.35 (*p* < 0.001) in younger Black individuals and 1.11 (*p* < 0.001) in middle-aged Black individuals, respectively [58]. However, the limited number of studies and moderate to high risk of bias resulted in low overall evidence quality for this association.

##### The predictive ability of anthropometric measures for T2D and their optimal cut-offs vary across ethnicities

Seven studies assessed the discriminative ability of T2D by anthropometric measures based on the receiver operating characteristics curve (ROC) and the area under the curve (AUC) [31, 37, 42, 51, 54, 59, 60]. Measures of central adiposity measures had higher ROC/AUC than overall obesity measures across ethnicities. Five studies suggested ethnicity-specific cut-offs of BMI, WC and WHR to identify high-risk populations for T2D, with lower thresholds for populations of East Asian, South Asian and Black ethnicities compared to people of White ethnicity [37, 59, 61–63] (Fig. S3a–c). The results of other anthropometric measures (skinfold thickness, the ratio of subscapular to triceps, hand grip strength, waist-to-hip-to-height ratio, hip circumference, a body shape index, body adiposity index, body height, trunk fat and trunk-to-leg ratio) with less evidence are shown in the Table S14. Due to the high proportion of cross-sectional studies and moderate/high risk of bias, these results have low/moderate evidence quality and should be interpreted cautiously.

##### Women-health associated factors

We found per one study for pregnancy index [64], breastfeeding [65], birth weight [66], and age at menarche [30]. Ethnic differences were noted in the relationships between pregnancy index, birth weight, age at menarche, and the risk of T2D, but not for breastfeeding, which was protective for all ethnic groups (Table S15). After adjustment for different confounders, a higher index of parity was associated with an elevated risk of T2D among White, South Asian and Chinese women, but Chinese women had a higher risk of T2D for 3-4 (13%) and ≥5 (359%) deliveries compared to women with 1 delivery than South Asian (3% and 23%) and White (13% and 52%) women during almost 12 years of follow-up [64]. Higher birth weight increased T2D odds for East Asian women but decreased it for White, Black, and Hispanic women [66]. Associations between breastfeeding, age at menarche, and T2D were non-significant in adjusted models [30, 65]. The quality of evidence for studies examining women’s health related factors was low.

The results for FHD are shown in Fig. S4 and Table S16.

## Discussion

This systematic review of 54 studies and meta-analysis of 12 studies summarized evidence on ethnic differences in the association between anthropometric measures, women-health-related factors, and T2D. Consistent with previous research, ethnic- and sex- differences in T2D risk were evident, and not always in a consistent direction. The median age of T2D diagnosis was 10 years earlier for South Asian individuals, seven years earlier for Black individuals, and five years earlier for East Asian compared to White individuals, suggesting that people of diverse ethnic backgrounds might benefit from earlier screening (<40 years) [15].

Our synthesis suggests that while BMI and weight gain are strongly associated with T2D in White groups, these measures are poorer predictors of T2D for people of South Asian, East Asian and Black ethnicities. Furthermore, we find that ratio measures of central adiposity such as WHR, and WHTR, may better predict T2D in all ethnic groups and both sexes, especially for people of Black ethnicity, for whom other anthropometric measures were less effective. BF % and WC were better predictors of T2D in women than in men, particularly those of White, Hispanic and South Asian ethnicities, but less so for those of Black ethnicity. However, WC’s effectiveness is influenced by height [37], making WHR and WHTR more reliable. As individuals’ height is relatively stable during adulthood, WHTR could better capture changes in WC, but our review identified fewer studies comparing its effect across ethnicities.

It is known that BMI does not reflect the proportions of lean and fat mass, where the contribution of the latter is more important in T2D development [37]. Moreover, it does not differentiate subcutaneous (SAT), visceral (VAT) adipose tissues and ectopic fat distributions, which are stronger associated with insulin resistance than general adiposity [42, 67]. Previous findings found that Asian individuals have more VAT and ectopic fat than people of White ethnicity, while people of Black ethnicity have less VAT compared to people of Asian and White ethnicities after adjustment to various total adiposity measures [42, 53, 68, 69]. Higher VAT is associated with enhanced proinflammatory markers and greater liver fat deposition leading to insulin resistance [70]. It was previously proposed that South Asians may have less capacity to store VAT at the same BMI levels, enhancing fat deposition in secondary depots such as the liver and pancreas [69, 71]. That means that changes in VAT may have more deteriorating effects on this group compared to white Europeans. However, despite having less VAT after controlling for BMI, people of Black ethnicity remained at a greater risk for T2D compared to those of White background [69]. This means different ethnicities may have distinct pathophysiology of T2D and contributing genetic susceptibility [34, 53]. It is still unclear if these ethnic differences occur due to variations in genetics, biological characteristics or lifestyle factors [9].

Therefore, relying solely on BMI might fail to identify high-risk ethnic groups or make weight-loss interventions ineffective for those with lower BMI but higher visceral fat. On the contrary, ratio measures of central adiposity may better represent changes in abdominal fat distribution, which are more strongly linked to insulin resistance [59]. Currently, the US Preventive Services Task Force (USPSTF) and American Diabetes Association (ADA) recommendations for earlier initiation of screening for T2D ( >35 years and any age) are mainly based on selecting individuals with overweight and obese BMI categories [16, 17]. Therefore, these guidelines may miss individuals of diverse backgrounds for whom BMI does not reflect T2D risk. Decreasing the T2D screening age for populations of diverse backgrounds and incorporating central adiposity measures into clinical practice could help to decrease health inequalities and improve timely preventive interventions. Since the quality of evidence coming predominantly from cross-sectional studies is low, more longitudinal research with a bigger population of younger ages studying the effect of changes in central obesity measures across ethnicities is needed.

This review demonstrated an emerging significance of sex-specific risk factors among women, even though the quality of evidence was low. East and South Asian women aged 18–50 had a higher T2D risk with increasing parity than White women in a Canadian study, with associations between parity and T2D strongest for Chinese women followed by South Asian and White women. Possible mechanisms could be mediated effect via postpartum weight retention and increased adiposity [64, 72]. However, a sensitivity analysis in this study showed that the attenuated but still significant effect remains even after adjustment for BMI, meaning parity may be an independent risk factor for diabetes. Ethnic differences in the effect of parity could be associated with differences in genetics. Previous studies demonstrated that Chinese women may have lower adiponectin levels in pregnancy, which is associated with postpartum insulin resistance and B-cell dysfunction [73, 74] and an increased risk of metabolic syndrome [75], while women of South Asian ethnicity may have other much stronger risk factors leading to an increased risk of T2D [64]. Another study revealed that after taking into account the histories of parity and breastfeeding, women with ≥5 parities had elevated T2D risk, regardless of breastfeeding duration, but adjusting for weight gain women with ≥3 parities did not have higher diabetes risk when they breastfed more than 12 months in total or each child more than 3 months [52]. Breastfeeding’s benefits might include higher energy expenditure and enhanced insulin sensitivity. Overall, breastfeeding was associated with reduced T2D risk across different ethnicities. Therefore, women with high parity potentially could be a target population for the prevention of T2D. More longitudinal studies which control for socio-economic variables and adiposity measures are needed to examine ethnic differences in the relationship between age at menarche, birth weight and T2D.

### Strengths and limitations

As only studies published in English were included, there is a potential for some findings from other non-English publications to be missed, which may result in a lack of findings from certain countries. However, it is difficult to predict how it might influence the review results. Secondly, we did not include studies focusing on environmental, clinical, genetic and lifestyle factors such as diet and physical activity which were out of the scope of this review, but which still contribute to the development of diabetes. Thirdly, most of the included studies were cross-sectional in design, which increases the potential risk of confounding, reverse causation, and bias. Future longitudinal studies are needed to validate these associations. Finally, meta-analyses were conducted based on a limited number of studies, largely due to inconsistencies in the units used for anthropometric measures across the included studies. In addition, comparisons between extreme BMI categories (e.g., BMI > 40 vs <25) may be less clinically meaningful than comparisons between more commonly used categories (e.g., BMI > 30 vs <25). Despite the small number of studies, the meta-analyses provide an important foundation for future research and help identify areas where more standardized and robust studies with clinically meaningful categorizations are needed. On the other hand, this systematic review has several strengths. Firstly, we did not restrict the search to certain clinical risk factors which allowed us to capture a broader number of studies. Secondly, the search strategy allowed us to identify evidence of ethnic differences in the effect of anthropometrics and the emerging importance of women-health-related factors on T2D for all ethnicities. In addition, this narrative synthesis allowed us to highlight the ethnic heterogeneity in the predictive ability of T2D using anthropometric measures, which are not currently considered in global multi-ethnic populations.

### Conclusion

This study demonstrated that ratio measures of central adiposity (WHR or WHTR) could be better than widely-used BMI at identifying people at high risk for T2D by sex and across ethnicities (particularly Asian and Black ethnic groups), with the importance of stratification of Asian ethnicities when assessing T2D risk. We have identified that the majority of evidence in this area is moderate/low and we recommend further high-quality longitudinal studies. Additionally, this systematic review highlighted the importance of ethnic- and sex-specific risk factors such as number of parities, birth weight and breastfeeding among women. These factors should be considered when assessing the risk of diabetes among women of South Asian, East Asian and Black ethnic backgrounds, which could be a target population for prevention.

## Supplementary information

Supplementary tables 3, 8-16

Supplementary tables (1-2, 4-7, 14-15) and figures (1-4)
