# Supplementary material for: Evidence of ethnic variations in the relationships between routinely recorded clinical factors and T2D: a systematic review and meta-analysis
Source: Int J Obes (Lond). 2025 Aug 11;49(10):1929–45. doi: 10.1038/s41366-025-01848-9 (PMC12532696; doi:10.1038/s41366-025-01848-9)
Supplement: Supplementary file 1 — Supplementary tables 3, 8-16 [file 41366_2025_1848_MOESM1_ESM.pdf]

Table S3. A table with results for studies including non-routine factors (or with insufficient evidence) on association with T2D.

Author, Year, Title	Study design	Country	Ethnic groups	Sample size, total and per each group	Number of cases (total, per ethnic group). T2D diagnosis	Data source	Participants' characteristics	Follow-up (years)	Risk factors	Results
**Jeong, 2021 (1)**	A longitudinal cohort study	Canada	South Asians, East Asians, Others	Total: 847 021. South Asians: 53,021. East Asians: 79,753. Others:714,247	Total:36,010 (cum, inc. 4.3%). T2D was defined at the occurrence of the second of two physician visits, within	British Columbia Hepatitis Testers Cohort (BT-HTC), 1990-2015	HCV negative: 55.3% females and 44.7% males. HCV positive: 35.2%	15 years of follow-up	**HCV infection**	HCV infection was associated with increased T2D risk in all ethnic groups: East Asians, adjusted HR (aHR) 3.07 (95%CI 2.43 to 3.88); South Asians, aHR 2.62 (95% CI 2.10 to 3.26); and Others, aHR 2.28 (95% CI 2.15 to 2.42). 'Other without HCV infection' was the reference group.
**Bank, 2017 (2)**	Cross-sectional study	Singapore and Sweden	Asian, White	Total: 20,539. Asian: 1,002; White: 19,537	Total: 5,249 (25.5%). Asian: 569 (56.7%); White: 4,680 (23.9%). FPG or HbA1c results or receiving antidiabetic therapy.	2 cohorts: the SHOP study (June 2010 and July 2014) from Singapore and the SwedeHF study (from	A cohort from Singapore: n=1,002, median [IQR] age 62 [54 to 70] years, 76% men, 19.5% obesity;	–	**Heart failure (HF), coronary artery disease (CAD)**	After multivariable adjustment, a stronger relationship between history of CAD and T2D was found in Asian patients compared to White patients (OR: 1.81 [95% CI: 1.30 to 2.52] vs. OR: 1.21 [95% CI: 1.11 to 1.31]; interaction p=0.014). Among patients with HF, there were no ethnic differences in the associations of diabetes with HF duration.
**Cabassa, 2011 (3)**	Cross-sectional study	USA	Non-Hispanic whites, African Americans, Hispanics,American [illegible]	Total: 34,653<br><br>Non-Hispanic whites: 20,161 African America [illegible]	NHW 7.61% (0.20), African Americans 11.52% (0.45), Hispanics 8.54% (0.54), [illegible]	Data were drawn from Wave 2 of the National Epidemiologic [illegible]	Men and women aged 18 years or older.	–	**Psychiatric disorders**	Hispanics, African Americans, AAI/AN, A/PI with psychiatric disorders compared to non-Hispanic Whites had higher risk of T2D than individuals without psychiatric disorders. For American Indians and Alaska Natives and Asians there were no difference in diabetes risk [illegible]
**Gurka, 2017 (4)**	Cohort study	USA	Black, White	Total: 13 094	Incidence of T2D: black women 17.1% and black men 16.8%, white men 11.7% and white women 8.0%. ARIC: self-report, FSG	Data from the ARIC study (n = 10,957) and the Jackson Heart Study (n = 2137)	In the ARIC study: white and black participants aged 45–64 years old (men and women). In the	7.8 years	**Metabolic syndrome (MS)**	There was no interaction between race and MS defined by ATP-III in the adjusted model. In the MS severity score model, there was a race × metabolic syndrome interaction such that HR was greater for black participants (5.30) than white participants (2.24).
**Wei, 2011 (5)**	Cohort study	USA	White, African American	Total: 10,893<br><br>Whites:8,388<br><br>African Americans:2,505	African Americans: 14.6%<br><br>Whites: 7.9%.<br><br>FBG or using	Data from ARIC, CARDIA, and the Framingham Heart Study	Men and women aged 34-54 years at baseline.	Median follow-up was 8.9 years	**Hypertension (normal, pre-hypertension, hypertension).**	Age-adjusted incidence was increasingly higher across increasing blood pressure groups (P-values for trend<0.05 for African American men;<0.001 for other race-sex groups). After multivariable adjustment prehypertension or hypertension (compared with

References

1. Jeong D, Karim ME, Wong S, Wilton J, Butt ZA, Binka M, Adu PA, Bartlett S, Pearce M, Clementi E, Yu A. Impact of HCV infection and ethnicity on incident type 2 diabetes: findings from a large population-based cohort in British Columbia. BMJ Open Diabetes Research and Care. 2021 Jun 1;9(1):e002145.
2. Bank IE, Gijsberts CM, Teng TH, Benson L, Sim D, Yeo PS, Ong HY, Jaufeerally F, Leong GK, Ling LH, Richards AM. Prevalence and clinical significance of diabetes in Asian versus white patients with heart failure. JACC: Heart Failure. 2017 Jan;5(1):14-24.
3. Cabassa LJ, Blanco C, Lopez-Castroman J, Lin KH, Lui SM, Lewis-Fernández R. Racial and ethnic differences in diabetes mellitus among people with and without psychiatric disorders: results from the National Epidemiologic Survey on Alcohol and Related Conditions. General hospital psychiatry. 2011 Mar 1;33(2):107-15.
4. Gurka MJ, Golden SH, Musani SK, Sims M, Vishnu A, Guo Y, Cardel M, Pearson TA, DeBoer MD. Independent associations between a metabolic syndrome severity score and future diabetes by sex and race: the Atherosclerosis Risk In Communities Study and Jackson Heart Study. Diabetologia. 2017 Jul;60(7):1261-70.
5. Wei GS, Coady SA, Goff Jr DC, Brancati FL, Levy D, Selvin E, Vasan RS, Fox CS. Blood pressure and the risk of developing diabetes in african americans and whites: ARIC, CARDIA, and the framingham heart study. Diabetes care. 2011 Apr 1;34(4):873-9.

Table S8. Trans-ethnic results of the effect of BMI on T2D from studies not included in the meta-analsysis (per SD or unit increment)

Study	Effect size	LCI	UCI	Ethnic group	Study design	Measure	Unit	Adjustment
Hardy, 2017a (men)	1.98	1.77	2.21	White	cross-sectional	OR	per unit increse (Z tranf.)	adjusted for age, physical activity, and FHD
Hardy, 2017a (women)	2	1.84	2.17	White	cross-sectional	OR	per unit increse (Z tranf.)	adjusted for age, physical activity, and FHD
Hardy, 2017b (men)	1.56	1.49	1.63	White	cohort	HR	per unit increse (Z tranf.)	Adjusted for age (5-year increment) over 4 visits
Hardy, 2017b (women)	1.76	1.68	1.84	White	cohort	HR	per unit increse (Z tranf.)	Adjusted for age (5-year increment) over 4 visits
Huxley, 2007 (men)	1.39	1.33	1.46	White	cross-sectional	OR	per 0.50 sd increase	
Huxley, 2007 (women)	1.32	1.28	1.37	White	cross-sectional	OR	per 0.50 sd increase	
Jenum, 2005 (men)	1.86	1.77	1.96	White	cross-sectional	OR	per SD increase	Adjusted for age, physical astivity, SES, body height and WHR
Jenum, 2005 (women	1.67	1.59	1.76	White	cross-sectional	OR	per SD increase	Adjusted for age, physical astivity, SES, body height and WHR
Kulick, 2017	1.64	1.27	2.04	White	cohort	HR	per SD increase	
Luo, 2018	1.45	1.43	1.47	White	cohort	HR	per SD increase	Adjusted for age at enrollment, education, FHD, lifestyle factors and cholesterol requiring medicine
Lutsey, 2010	1.99	1.73	2.32	White	cohort	HR	per SD increase	Adjusted for age, sex, race/ethnicity, education, and income.
Mackay, 2010	2.22	1.63	3.02	White	cohort	OR	per SD increase	Adjusted for age and sex
Marshall, 1993	1.28	1.16	1.44	White	case-control	OR	per unit increase	Adjusted for age and sex
Resnick, 1998 (men)	1.22	1.18	1.25	White	cohort	OR	per unit increase	Adjusted for age, education and subscapular-to-triceps skinfold ratio
Resnick, 1998 (women)	1.2	1.16	1.23	White	cohort	OR	per unit increase	Adjusted for age, education and subscapular-to-triceps skinfold ratio
Zamoora, 2019	1.12	1.09	1.16	White	cohort	RR	per 5 percentile	
Jenum, 2005 (men)	2.11	1.82	2.45	South Asian	cross-sectional	OR	per SD increase	Adjusted for age, physical astivity, SES, body height and WHR
Jenum, 2005 (women)	1.22	1.09	1.36	South Asian	cross-sectional	OR	per SD increase	Adjusted for age, physical astivity, SES, body height and WHR
Nan, 2008 (men)	1.41	1.26	1.58	South Asian	cohort	HR	per SD increase	adjusted for uric acid, TG, FPG
Nan, 2008 (women)	1.32	1.17	1.49	South Asian	cohort	HR	per SD increase	adjusted for uric acid, TG, FPG
Narayan, 2021	1.05	1.02	1.08	South Asian	cohort	HR	per unit increase	Adjusted for age, sex, FHD, log HOMA-IR, log HOMA-B
Luo, 2018	1.56	1.24	1.95	Others	cohort	HR	per SD increase	Adjusted for age at enrollment, education, FHD, lifestyle factors and cholesterol requiring medicine
Zamoora, 2019	1.09	1.02	1.17	Others	cohort	RR	per 5 percentile	
Kulick, 2017	1.37	1.22	1.55	Hispanic	cohort	HR	per SD increase	
Luo, 2018	1.39	1.32	1.46	Hispanic	cohort	HR	per SD increase	Adjusted for age at enrollment, education, FHD, lifestyle factors and cholesterol requiring medicine
Mackay, 2010	1.62	1.2	2.17	Hispanic	cohort	OR	per SD increase	Adjusted for age and sex
Marshall, 1993	1.26	1.14	1.38	Hispanic	case-control	OR	per unit increase	Adjusted for age and sex
Zamoora, 2019	1.12	1.06	1.18	Hispanic	cohort	RR	per 5 percentile	
Huxley, 2007 (men)	1.26	1.2	1.33	East Asian	cross-sectional	OR	per 0.50 sd increase	
Huxley, 2007 (women)	1.23	1.19	1.28	East Asian	cross-sectional	OR	per 0.50 sd increase	
Luo, 2018	1.51	1.39	1.63	East Asian	cohort	HR	per SD increase	Adjusted for age at enrollment, education, FHD, lifestyle factors and cholesterol requiring medicine
Hardy, 2017a (men)	1.77	1.53	2.04	ck/African Caribbe	cross-sectional	OR	per unit increse (Z tranf.)	adjusted for age, physical activity, and FHD
Hardy, 2017a (men)	1.4	1.29	1.52	ck/African Caribbe	cross-sectional	OR	per unit increse (Z tranf.)	adjusted for age, physical activity, and FHD
Hardy, 2017b (men)	1.51	1.41	1.63	ck/African Caribbe	cohort	HR	per unit increse (Z tranf.)	Adjusted for age (5-year increment) over 4 visits
Hardy, 2017b (men)	1.43	1.35	1.52	ck/African Caribbe	cohort	HR	per unit increse (Z tranf.)	Adjusted for age (5-year increment) over 4 visits
Kulick, 2017	1.07	0.85	1.35	ck/African Caribbe	cohort	HR	per SD increase	
Luo, 2018	1.29	1.25	1.33	ck/African Caribbe	cohort	HR	per SD increase	Adjusted for age at enrollment, education, FHD, lifestyle factors and cholesterol requiring medicine
Mackay, 2010	1.46	1.04	2.03	ck/African Caribbe	cohort	OR	per SD increase	Adjusted for age and sex
Nan, 2008 (men)	1.69	1.4	2.05	ck/African Caribbe	cohort	HR	per SD increase	adjusted for uric acid, TG, FPG
Nan, 2008 (women)	1.33	1.12	1.58	ck/African Caribbe	cohort	HR	per SD increase	adjusted for uric acid, TG, FPG
Resnick, 1998 (men)	1.14	1.08	1.2	ck/African Caribbe	cohort	OR	per unit increase	Adjusted for age, education and subscapular-to-triceps skinfold ratio
Resnick, 1998 (women)	1.16	1.11	1.19	ck/African Caribbe	cohort	OR	per unit increase	Adjusted for age, education and subscapular-to-triceps skinfold ratio
Zamoora, 2019	1.06	1.04	1.09	ck/African Caribbe	cohort	RR	per 5 percentile	

Table S9. Trans-ethnic results of the effect of BMI on T2D from studies not included in the meta-analsysis (categorical)

Study	Effect size	LCI	UCI	Ethnic group	Study design	Measure	Unit	Adjustment
Chan, 2018	1.53	1	2.33	South Asian	cohort	RR	overweight vs. underweight or norma	Adjusted for age, gender, ethnicity, family history of diabetes, income, education, current smoking status, systolic blood pressure, HbA1c, total cholesterol, HDL cholesterol
Chan, 2018	2.05	1.28	3.28	South Asian	cohort	RR	obese vs. underweight or normal	Adjusted for age, gender, ethnicity, family history of diabetes, income, education, current smoking status, systolic blood pressure, HbA1c, total cholesterol, HDL cholesterol
Chan, 2018	1.86	1.3	2.65	South Asian	cohort	RR	overweight vs. underweight or norma	Adjusted for age, gender, ethnicity, family history of diabetes, income, education, current smoking status, systolic blood pressure, HbA1c, total cholesterol, HDL cholesterol
Chan, 2018	2.02	1.28	3.2	South Asian	cohort	RR	obese vs. underweight or normal	Adjusted for age, gender, ethnicity, family history of diabetes, income, education, current smoking status, systolic blood pressure, HbA1c, total cholesterol, HDL cholesterol
Li, 2017	7.17	1.85	27.75	Others	cross-sectional	OR	overweight vs. normal	Adjusted for mediacaid status, sex, age, marital status, checkup status, physical activity, immunization, alcohol consumption
Li, 2018	7.45	2.06	26.96	Others	cross-sectional	OR	obese vs. normal	Adjusted for mediacaid status, sex, age, marital status, checkup status, physical activity, immunization, alcohol consumption
Luo, 2018	1.45	0.72	2.94	American Indian	cohort	HR	overweight vs. normal	Adjusted for age at enrollment, education, FHD, lifestyle factors and cholesterol requiring medicine
Luo, 2018	3.64	1.86	7.15	American Indian	cohort	HR	obese vs. normal	Adjusted for age at enrollment, education, FHD, lifestyle factors and cholesterol requiring medicine
Luo, 2018	2.2	1.79	3.38	East Asian	cohort	HR	overweight vs. normal	Adjusted for age at enrollment, education, FHD, lifestyle factors and cholesterol requiring medicine
Luo, 2018	3.38	2.55	4.46	East Asian	cohort	HR	obese vs. normal	Adjusted for age at enrollment, education, FHD, lifestyle factors and cholesterol requiring medicine
Luo, 2018	1.65	1.41	1.92	Black/African American	cohort	HR	overweight vs. normal	Adjusted for age at enrollment, education, FHD, lifestyle factors and cholesterol requiring medicine
Luo, 2018	2.57	2.22	2.97	Black/African American	cohort	HR	obese vs. normal	Adjusted for age at enrollment, education, FHD, lifestyle factors and cholesterol requiring medicine
Luo, 2018	1.82	1.47	2.25	Hispanic	cohort	HR	overweight vs. normal	Adjusted for age at enrollment, education, FHD, lifestyle factors and cholesterol requiring medicine
Luo, 2018	3.41	2.76	4.22	Hispanic	cohort	HR	obese vs. normal	Adjusted for age at enrollment, education, FHD, lifestyle factors and cholesterol requiring medicine
Ma, 2012	2.94	2.78	3.03	White	cohort	HR	overweight/obese vs. normal	Adjusted for age, family history of diabetes, hormone therapy use, study arm, and each lifestyle risk factors
Ma, 2012	2.22	1.88	2.63	Black/African American	cohort	HR	overweight/obese vs. normal	Adjusted for age, family history of diabetes, hormone therapy use, study arm, and each lifestyle risk factors
Ma, 2012	2.78	2.17	3.45	Hispanic	cohort	HR	overweight/obese vs. normal	Adjusted for age, family history of diabetes, hormone therapy use, study arm, and each lifestyle risk factors
Ma, 2012	2.94	2.38	3.7	East Asian	cohort	HR	overweight/obese vs. normal	Adjusted for age, family history of diabetes, hormone therapy use, study arm, and each lifestyle risk factors
Zhu, 2019	1.94	1.92	1.97	Hispanic	cross-sectional	OR	overweight vs. normal	Adjusted for age, sex, neighborhood education, neighborhood poverty level
Zhu, 2019	2.65	2.61	2.69	Hispanic	cross-sectional	OR	obese class 1 vs normal	Adjusted for age, sex, neighborhood education, neighborhood poverty level
Zhu, 2019	3.43	3.38	3.49	Hispanic	cross-sectional	OR	obese class 2 vs normal	Adjusted for age, sex, neighborhood education, neighborhood poverty level
Zhu, 2019	3.43	3.38	3.49	Hispanic	cross-sectional	OR	obese class 3 vs normal	Adjusted for age, sex, neighborhood education, neighborhood poverty level
Zhu, 2019	4.53	4.41	4.67	Hispanic	cross-sectional	OR	obese class 4 vs normal	Adjusted for age, sex, neighborhood education, neighborhood poverty level
Zhu, 2019	1.8	1.73	1.88	Hawaiian/Pacific Islander	cross-sectional	OR	overweight vs. normal	Adjusted for age, sex, neighborhood education, neighborhood poverty level
Zhu, 2019	2.29	2.19	2.39	Hawaiian/Pacific Islander	cross-sectional	OR	obese class 1 vs normal	Adjusted for age, sex, neighborhood education, neighborhood poverty level
Zhu, 2019	2.75	2.62	2.88	Hawaiian/Pacific Islander	cross-sectional	OR	obese class 2 vs normal	Adjusted for age, sex, neighborhood education, neighborhood poverty level
Zhu, 2019	2.75	2.62	2.88	Hawaiian/Pacific Islander	cross-sectional	OR	obese class 3 vs normal	Adjusted for age, sex, neighborhood education, neighborhood poverty level
Zhu, 2019	3.19	2.95	3.45	Hawaiian/Pacific Islander	cross-sectional	OR	obese class 4 vs normal	Adjusted for age, sex, neighborhood education, neighborhood poverty level
Zhu, 2019	2.27	2.08	2.48	merican Indian/Alaska Nativ	cross-sectional	OR	overweight vs. normal	Adjusted for age, sex, neighborhood education, neighborhood poverty level
Zhu, 2019	3.09	2.82	3.39	merican Indian/Alaska Nativ	cross-sectional	OR	obese class 1 vs normal	Adjusted for age, sex, neighborhood education, neighborhood poverty level
Zhu, 2019	3.73	3.38	4.11	merican Indian/Alaska Nativ	cross-sectional	OR	obese class 2 vs normal	Adjusted for age, sex, neighborhood education, neighborhood poverty level
Zhu, 2019	3.73	3.38	4.11	merican Indian/Alaska Nativ	cross-sectional	OR	obese class 3 vs normal	Adjusted for age, sex, neighborhood education, neighborhood poverty level
Zhu, 2019	4.85	4.24	5.55	merican Indian/Alaska Nativ	cross-sectional	OR	obese class 4 vs normal	Adjusted for age, sex, neighborhood education, neighborhood poverty level

Table S10. Trans-ethnic results of the effect of WHR on T2D

Study	Effect size	LCI	UCI	Ethnic group	Study design	Measure	Unit
Hardy, 2017b (men)	1.26	1.08	1.47	White	cohort	HR	per SD increase (log-scale)
Hardy, 2017b (women)	1.72	1.45	2.05	White	cohort	HR	per unit increase (z-scale)
Huxley, 2007 (men)	1.41	1.33	1.5	White	cross-sectional	OR	per 0.5 SD increase
Huxley, 2007 (women)	1.62	1.52	1.72	White	cross-sectional	OR	per 0.5 SD increase
Jenum, 2005 (men)	2.3	1.68	3.16	White	cross-sectional	OR	per 0.5 SD increase
Jenum, 2005 (women)	2.49	1.76	3.5	White	cross-sectional	OR	per 0.5 SD increase
Luo, 2018	1.22	1.21	1.23	White	cohort	HR	per SD increase
MacKay, 2010	2.19	1.39	3.45	White	cohort	OR	SD change in log-tranf.
Marshall, 1993	2.1	1.4	3.2	White	case-control	OR	per 0.1 unit increase
Steinbrecher, 2015 (men)	1.32	1.22	1.43	White	cross-sectional	OR	per SD increase
Steinbrecher, 2015 (women	1.73	1.55	1.93	White	cross-sectional	OR	per SD increase
Abdullah, 2018	3.01	2.3	3.93	South Asian	case-control	OR	per unit increase
Abdullah, 2018	3.23	2.46	4.23	South Asian	case-control	OR	per unit increase
Jenum, 2005 (men)	3.95	1.4	11.17	South Asian	cross-sectional	OR	per SD increase
Jenum, 2005 (women)	2.21	1.06	4.59	South Asian	cross-sectional	OR	per SD increase
Luo, 2018	1.25	1.07	1.46	Others	cohort	HR	per SD increase
Steinbrecher, 2015 (men)	1.22	1.09	1.36	Native Hawaiian	cross-sectional	OR	per SD increase
Steinbrecher, 2015 (women	1.4	1.26	1.56	Native Hawaiian	cross-sectional	OR	per SD increase
Steinbrecher, 2015 (men)	1.22	1.14	1.29	Japanese American	cross-sectional	OR	per SD increase
Steinbrecher, 2015 (women	1.48	1.39	1.58	Japanese American	cross-sectional	OR	per SD increase
Luo, 2018	1.23	1.19	1.27	Hispanic	cohort	HR	per SD increase
MacKay, 2010	1.38	0.9	2.13	Hispanic	cohort	OR	SD change in log-tranf.
Marshall, 1993	1.3	0.9	1.9	Hispanic	case-control	OR	per 0.1 unit increase
Abdullah, 2018	6.28	4.27	9.25	East Asian	case-control	OR	per unit increase
Huxley, 2007 (men)	1.47	1.35	1.6	East Asian	cross-sectional	OR	per 0.5 SD increase
Huxley, 2007 (women)	1.4	1.29	1.52	East Asian	cross-sectional	OR	per 0.5 SD increase
Luo, 2018	1.19	1.15	1.24	East Asian	cohort	HR	per SD increase
Luo, 2018	1.25	1.22	1.29	Black	cohort	HR	per SD increase
Hardy, 2017b (men)	1.63	1.43	1.86	Black	cohort	HR	per unit increase (z-scale)
Hardy, 2017b (women)	1.77	1.59	1.97	Black	cohort	HR	per unit increase (z-scale)
MacKay, 2010	2.52	1.57	4.05	Black	cohort	OR	per SD increase (log-scale)

Table S11. Trans-ethnic results of the effect of WC on T2D

Study	Effect size	LCI	UCI	Ethnic group	Study design	Measure	Unit
Hardy, 2017a (men)	2.02	1.8	2.27	White	cross-sectional	OR	per unit increase (Z score)
Hardy, 2017a (women)	2.16	1.98	2.35	White	cross-sectional	OR	per unit increase (Z score)
Huxley, 2007 (men)	1.42	1.36	1.5	White	cross-sectional	OR	per 0.5 SD increase
Huxley, 2007 (women)	1.5	1.44	1.58	White	cross-sectional	OR	per 0.5 SD increase
MacKay, 2010	2.25	1.59	3.17	White	cohort	OR	per SD increase (log-scale)
Luo, 2018	1.55	1.53	1.57	White	cohort	HR	per SD increase (log-scale)
Hardy, 2017b (men)	1.56	1.5	1.63	White	cohort	HR	per unit increase (Z score)
Hardy, 2017b (women)	1.88	1.78	1.98	White	cohort	HR	per unit increase (Z score)
Bennet, 2014	1.05	0.99	1.12	White	cross-sectional	OR	per SD increase
Steinbrecher, 2015 (women)	1.99	1.79	2.2	White	cross-sectional	OR	per SD increase
Steinbrecher, 2015 (men)	1.63	1.5	1.77	White	cross-sectional	OR	per SD increase
Luo, 2018	1.57	1.29	1.91	Others	cohort	HR	per SD increase
Steinbrecher, 2015 (women)	1.62	1.47	1.78	Native Hawaiian	cross-sectional	OR	per SD increase
Steinbrecher, 2015 (men)	1.36	1.23	1.5	Native Hawaiian	cross-sectional	OR	per SD increase
Steinbrecher, 2015 (women)	1.87	1.74	2.02	Japanese American	cross-sectional	OR	per SD increase
Steinbrecher, 2015 (men)	1.54	1.44	1.65	Japanese American	cross-sectional	OR	per SD increase
Bennet, 2014	1.05	1.01	1.1	Iraqi	cross-sectional	OR	per SD increase
MacKay, 2010	1.59	1.15	2.19	Hispanic	cohort	OR	per SD increase (log-scale)
Luo, 2018	1.58	1.5	1.67	Hispanic	cohort	HR	per SD increase
Huxley, 2007 (men)	1.35	1.28	1.43	East Asian	cross-sectional	OR	per 0.5 SD increase
Huxley, 2007 (women)	1.4	1.32	1.47	East Asian	cross-sectional	OR	per 0.5 SD increase
Luo, 2018	1.93	1.76	2.12	East Asian	cohort	HR	per SD increase
Hardy, 2017a (men)	1.78	1.55	2.06	Black /African Caribbean	cross-sectional	OR	per unit increase (Z score)
Hardy, 2017a (women)	1.61	1.47	1.76	Black /African Caribbean	cross-sectional	OR	per unit increase (Z score)
MacKay, 2010	1.51	1.08	2.11	Black /African Caribbean	cohort	OR	per SD increase (log-scale)
Luo, 2018	1.42	1.37	1.47	Black /African Caribbean	cohort	HR	per SD increase
Hardy, 2017b (men)	1.57	1.45	1.7	Black /African Caribbean	cohort	HR	per unit increase (Z score)
Hardy, 2017b (women)	1.54	1.44	1.65	Black /African Caribbean	cohort	HR	per unit increase (Z score)

Table S12. Trans-ethnic results of the effect of WHTR on T2D

Study	Effect size	LCI	UCI	Ethnic group	Study design	Measure	Unit
Hardy, 2017a (men)	2.19	1.94	2.48	White	cross-sectional	OR	per unit increase (z-score)
Hardy, 2017a (women)	2.09	1.92	2.27	White	cross-sectional	OR	per unit increase (z-score)
Hardy, 2017b (men)	1.57	1.5	1.64	White	cohort	HR	per unit increase (z-score)
Hardy, 2017b (women)	1.86	1.76	1.96	White	cohort	HR	per unit increase (z-score)
MacKay, 2010	2.25	1.63	3.1	White	cohort	OR	per SD increase
Steinbrecher, 2015 (mer	1.69	1.56	1.84	White	cross-sectional	OR	per SD increase
Steinbrecher, 2015 (wor	2.04	1.84	2.26	White	cross-sectional	OR	per SD increase
Steinbrecher, 2015 (mer	1.37	1.24	1.51	Native Hawaiian	cross-sectional	OR	per SD increase
Steinbrecher, 2015 (wor	1.6	1.45	1.76	Native Hawaiian	cross-sectional	OR	per SD increase
Steinbrecher, 2015 (mer	1.48	1.39	1.59	Japanese	cross-sectional	OR	per SD increase
Steinbrecher, 2015 (wor	1.8	1.68	1.93	Japanese	cross-sectional	OR	per SD increase
MacKay, 2010	1.55	1.14	2.1	Hispanic	cohort	OR	per SD increase
Hardy, 2017a (men)	1.92	1.65	2.25	Black	cross-sectional	OR	per unit increase (z-score)
Hardy, 2017a (women)	1.57	1.44	1.71	Black	cross-sectional	OR	per unit increase (z-score)
Hardy, 2017b (men)	1.54	1.42	1.66	Black	cohort	HR	per unit increase (z-score)
Hardy, 2017b (women)	1.54	1.44	1.65	Black	cohort	HR	per unit increase (z-score)
MacKay, 2010	1.62	1.16	2.26	Black	cohort	OR	per SD increase

Table S13. Trans-ethnic results of the effect of body fat percentage on T2D

Study	Effect size	LCI	UCI	Ethnic group	Study design	Measure
Meeks, 2015 (men)	1.08	1.02	1.14	White	cross-sectional	OR
Meeks, 2015 (women)	1.18	1.09	1.27	White	cross-sectional	OR
Luo, 2019	1.38	1.29	1.48	White	cohort	HR
MacKay, 2010	3.43	2.06	5.71	White	cohort	OR
Luo, 2019	1.4	1.09	1.8	Hispanic	cohort	HR
MacKay, 2010	2.12	1.3	3.38	Hispanic	cohort	OR
Meeks, 2015 (men)	1.04	1	1.08	Black	cross-sectional	OR
Meeks, 2015 (men)	1.09	1.04	1.13	Black	cross-sectional	OR
Meeks, 2015 (women)	1.03	0.99	1.07	Black	cross-sectional	OR
Meeks, 2015 (women)	1.09	1.05	1.13	Black	cross-sectional	OR
Luo, 2019	1.23	1.07	1.41	Black	cohort	HR
MacKay, 2010	1.49	0.88	2.54	Black	cohort	OR

**Unit**
per unit increase (centered)
per unit increase (centered)
per unit increase
per SD increase
per unit increase
per SD increase
per unit increase (centered)
per unit increase (centered)
per unit increase (centered)
per unit increase (centered)
per unit increase
per SD increase

Table S16. Trans-ethnic results of the effect of family history of diabetes on T2D

Study	Effect size	LCI	UCI	Ethnic group	Study design	Measure
Zamora-Kapoor, 2018	1.85	1.22	2.8	White	cohort	RR
Marshall, 1993	5.4	3.2	9.4	White	case-control	OR
Narayan, 2021	1.53	1.35	1.75	White	cohort	HR
Abdullah, 2018	0.77	0.52	1.12	South Asian	case-control	OR
Abdullah, 2018	1.64	1.13	2.38	South Asian	case-control	OR
Narayan, 2021	1.23	0.86	1.75	South Asian	cohort	HR
Zamora-Kapoor, 2018	2.4	1.5	3.86	Hispanic	cohort	RR
Marshall, 1993	4.1	2.5	6.5	Hispanic	case-control	OR
Abdullah, 2018	1.9	1.22	2.96	East Asian	case-control	OR
Narayan, 2021	1.37	1.14	1.65	Black	cohort	HR
