# Supplementary material for: Evidence of ethnic variations in the relationships between routinely recorded clinical factors and T2D: a systematic review and meta-analysis
Source: Int J Obes (Lond). 2025 Aug 11;49(10):1929–45. doi: 10.1038/s41366-025-01848-9 (PMC12532696; doi:10.1038/s41366-025-01848-9)
Supplement: Supplementary file 2 — Supplementary tables (1-2, 4-7, 14-15) and figures (1-4) [file 41366_2025_1848_MOESM2_ESM.pdf]

Supplementary materials, methods and results

Table S1. MESH terms and keywords used in the Medline Complete database.

**Medline**	TI ((MH "Diabetes Mellitus, Type 2+") OR (MH "Diabetes Mellitus+") OR "type 2 diabetes" OR "type II diabetes" OR "diabetes mellitus") AND TI ("ethnicity" OR "Ethnic minority" OR "Multi-ethnic" OR "Ethnic" OR (MH "Ethnic and Racial Minorities") OR (MH "Ethnicity") OR "race-ethnic" OR "race" OR "Chinese" OR "Arab" OR "Asian" OR "African" OR "Caribbean" OR "Black").<br>Filters applied: human, English, 1990-2023, Journal article, Medline database

Table S2. Categorisation of ethnicities from included studies into higher-level ethnicities

**South Asian**	• Indian/ American Indian/ Asian Indian/ Hindustani Surinamese<br>• Pakistani<br>• Bangladeshi<br>• Sri Lankan<br>• South Asian/ South Asian Surinamese
**Black**	• Black/ Non-Hispanic Black/ Black American/ African-Caribbean/ African Surinamese<br>• Jamaican<br>• Nigerian<br>• Mauritius Creoles<br>• Ghanian
**East Asian**	• East Asian<br>• Korean<br>• Japanese/ Japanese American<br>• Chinese/ Han Chinese/ Chinese American

	• Uyghur
**Southeast Asian**	• Filipino<br>• Vietnamese<br>• Malay
**White**	• White/ US White/ UK White/ Non-Hispanic White<br>• European<br>• Norway<br>• Dutch
**Hispanic**	• Hispanics/ Hispanic American<br>• Mexican/ Mexican American<br>• Latino/ Latin American
**Middle East/ Arab**	• Arab<br>• United Arab Emirates<br>• Iraq<br>• Moroccan<br>• Turkish
**Others**	• Hawaiian<br>• Aboriginal<br>• Other Indigenous<br>• American Indians/Alaska Natives<br>• Pacific Islanders<br>• Mauritian<br>• Asian/Asian American

Table S3. A table with results for five single risk factor studies (Supplementary excel file)

Table S4. Number of studies per anthropometric measures and unit measures.

Anthropometric measure	Number of studies considered	Ethnicities represented
**BMI (categorical)**	9	South Asian, East Asian, White, Others, American Indian, Black/African Caribbean, Hispanic

**BMI (per SD or linear)**	15	Black/African Caribbean, White, Hispanic, East Asian, South Asian, American Indian, Others
**Weight gain (categorical)**	2	White, Black/African Caribbean, Hawaiian, Japanese American
**Weight gain (per 1 log-increase in BMI-year)**	1	White, Black/African Caribbean
**Body fat percentage (per SD or linear)**	5	Black/African Caribbean, White, Hispanic, South Asian, East Asian, Turkish, Moroccan
**Trunk fat, trunk-to-leg-ratio (per quartile and linear/unit)**	1	Black/African Caribbean, Hispanic
**WC (per SD or linear)**	9	Asian Indian, Mauritian Indian, East Asian, White, Iraqi, Black/African Caribbean, Turkish, Southeast Asian, Hispanic, Others
**WC (categorical)**	1	Jamaican, U.S.
**WHR (per SD or linear)**	11	South Asian, East Asian, Southeast Asian, White, Black/African Caribbean, Hispanic, Native Hawaiian, Japanese American, Turkish, Moroccan, Others
**WHTR (per SD and linear)**	4	Black/African Caribbean, White, Hispanic, Native Hawaiian, Japanese
**WHHR (per unit/linear)**	1	White, Black/African Caribbean
**Body height (per unit/linear [cm])**	1	White, South Asian
**Skinfold thickness/Triceps skinfold/Subscapular skinfold/Sum of skinfold thickness (per SD or linear)**	2	White, Hispanic, Black/African Caribbean/African American
**The ratio of subscapular to triceps (per unit/linear)**	2	White, Hispanic, Black/African Caribbean/African American
**Hand grip strength (per SD)**	1	White, Black/African Caribbean, South Asian
**Hip circumference (per SD)**	2	White, Native Hawaiian, Japanese American, Black/African Caribbean, Hispanic

**ABSI (per SD)**	2	White, Black/African Caribbean
**BAI (per SD)**	2	White, Black/African Caribbean
**Birth weight (categorical)**	1	White, Black/African Caribbean, Asian/Pacific Islander, Hispanic
**Combination of anthropometric measures**	2	White, Native Hawaiian, Japanese American, Black, Aboriginal, South Asian, Hispanic

Table S5. Risk of bias assessment. NOS scale for case-control studies

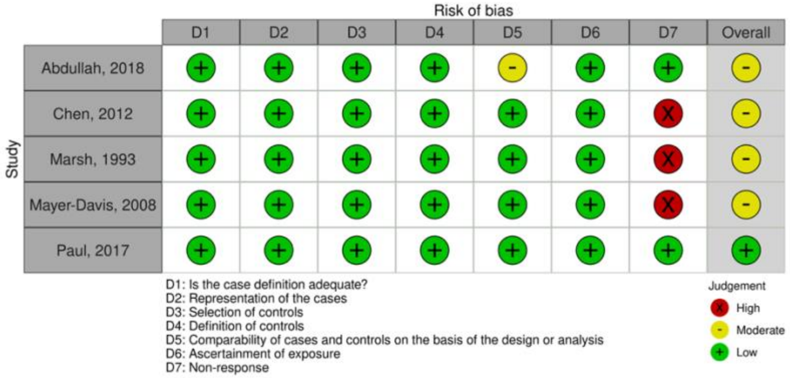

.

Table S6. Risk of bias assessment. NOS scale for cohort studies.

Study	D1	D2	D3	D4	D5	D6	D7	Overall
Caleyachetty, 2021	+	+	+	+	+	+	+	+
Dreydus, 2012	+	+	+	+	+	+	+	+
Hardy, 2017	+	+	+	+	+	+	+	+
Jeong, 2021	+	+	+	+	+	+	+	+
Mackay, 2009	+	+	+	+	+	+	+	+
Almahmeed, 2017	+	+	+	+	+	+	+	+
Chan, 2018	+	+	+	+	+	+	+	+
Chatterjee, 2016	+	+	+	+	+	-	+	-
Chiu, 2011	+	+	X	+	+	+	+	-
Gurk, 2017	+	+	+	X	+	+	+	-
Kulick, 2016	+	+	+	X	+	+	+	-
Luo, 2019	+	+	+	+	+	+	+	+
Lutsey, 2010	+	+	+	+	+	+	+	+
Ma, 2012	+	+	+	+	+	+	+	+
Maskarinec, 2009	+	+	+	+	-	+	+	-
Morimoto, 2011	+	+	+	+	-	+	+	-
Nan, 2008	+	+	+	+	+	+	+	+
Narayan, 2021	+	+	+	+	+	+	+	+
Piccolo, 2014	+	+	+	+	+	-	+	-
Piccolo, 2013	X	+	+	+	+	+	+	-
Reis, 2016	+	+	+	+	+	+	+	+
Resnick, 1998	+	+	+	-	+	+	+	-
Rodriguez, 2021	+	+	+	-	+	+	+	-
Shaten, 1993	+	+	+	+	+	+	+	+
Stevens, 2008	+	+	-	+	+	+	+	-
Tillin, 2013	+	+	+	+	+	+	+	+
Tillin, 2014	+	+	+	+	+	-	+	-
Wei, 2011	+	+	+	+	+	+	+	+
Wei, 2015	+	+	+	+	+	+	+	+
Zamora-Kapoor, 2018	+	+	+	+	+	+	+	+
Beihl, 2009	+	+	+	+	+	+	+	+

Risk of bias

D1: Representativeness of the exposed cohort
D2: Selection of the non-exposed cohort
D3: Ascertainment of the exposure
D4: Demonstration that outcome of interest was not present at the start of study
D5: Comparability of the cohorts on the basis of the design or analysis
D6: Ascertainment of exposure
D7: Non-response rate

Judgement: X High; - Moderate; + Low

Table S7. JBI critical appraisal checklist for cross-sectional studies

Study	D1	D2	D3	D4	D5	D6	D7	D8	Overall
Alperet, 2016	+	+	X	+	X	X	+	-	X
De Koning, 2010	+	+	+	+	+	+	+	+	+
Hardy, 2017	+	+	+	+	+	+	+	+	+
Meeks, 2015	+	+	+	+	X	-	+	+	-
Aggarwal, 2022	+	+	+	+	X	-	+	+	-
Bennet, 2014	+	+	+	+	+	+	+	+	+
Cheong, 2014	+	+	+	+	X	-	+	+	-
Cheong, 2013	+	+	+	+	X	-	+	+	-
Cohen, 2009	?	+	X	X	+	+	X	+	X
Diaz, 2007	+	+	+	+	X	-	+	+	-
Gong, 2015	+	+	+	+	+	+	+	+	+
Hamoudi, 2019	+	+	+	+	X	-	+	X	X
Huxley, 2008	X	?	+	+	X	-	+	+	X
Jenum, 2012	+	+	+	+	+	+	+	+	+
Jenum, 2005	+	+	+	+	+	+	+	+	+
Li, 2017	+	+	X	X	+	+	-	+	X
Lorenzo, 2007	+	X	+	+	X	-	+	+	X
Ntuk, 2017	+	+	+	+	+	+	+	+	+
Ntuk, 2014	+	+	+	+	+	+	+	+	+
Okosun, 1998	+	+	+	+	X	X	+	+	-
Ryckman, 2014	+	+	X	+	+	X	+	+	-
Strings, 2023	X	X	+	+	X	X	+	+	X
Vicks, 2022	+	+	+	+	+	X	+	+	+
Yoon, 2015	+	+	+	+	+	+	+	+	+
Zethof, 2021	+	+	+	+	X	X	+	+	-
Signorello, 2007	+	+	+	+	+	+	+	X	+
Steinbrecher, 2015	+	+	+	+	+	+	+	X	+
Nyamdorj, 2010	+	+	+	+	+	+	+	+	+
Zhu, 2019	+	+	+	+	+	+	+	+	+

Risk of bias

D1: Were the criteria for inclusion in the sample clearly defined?
D2: Were the study subjects and the setting described in detail?
D3: Was the exposure measured in a valid and reliable way?
D4: Were objective, standard criteria used for measurement of the condition?
D5: Were confounding factors identified?
D6: Were strategies to deal with confounding factors stated?
D7: Were the outcomes measured in a valid and reliable way?
D8: Was appropriate statistical analysis used?

Judgement: X High; - Unclear; + Low; ? No information

Figure S1a-b. Trans-ethnic meta-analysis of the effect of BMI ((a) categorical and (b) per unit increase, HR)

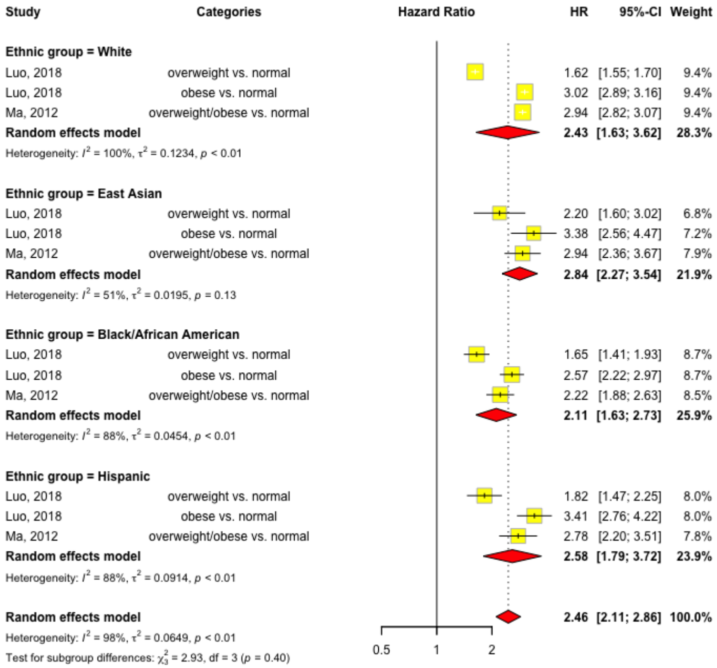

Figure S1a.

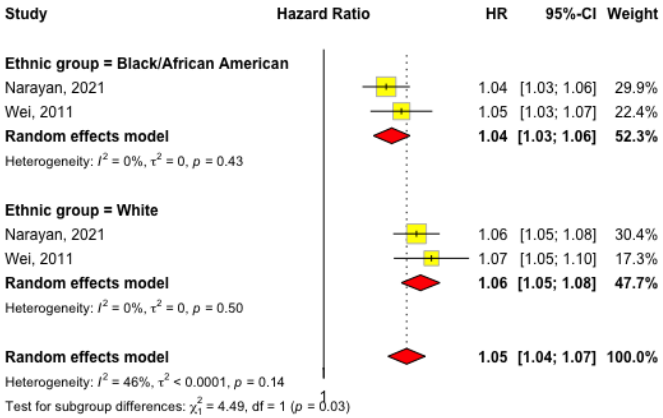

Figure S1b.

Table S8. Trans-ethnic results of the effect of BMI on T2D from studies not included in the meta-analysis (per SD or unit increase) (Supplementary excel file)

Table S9. Trans-ethnic results of the effect of BMI on T2D from studies not included in the meta-analysis (categorical) (Supplementary excel file)

Table S10. Trans-ethnic results of the effect of WHR on T2D (Supplementary excel file)

Table S11. Trans-ethnic results of the effect of WC on T2D (Supplementary excel file)

Table S12. Trans-ethnic results of the effect of WHTR on T2D (Supplementary excel file)

Table S13. Trans-ethnic results of the effect of body fat percentage on T2D (Supplementary excel file)

Figure S2. Funnel plots

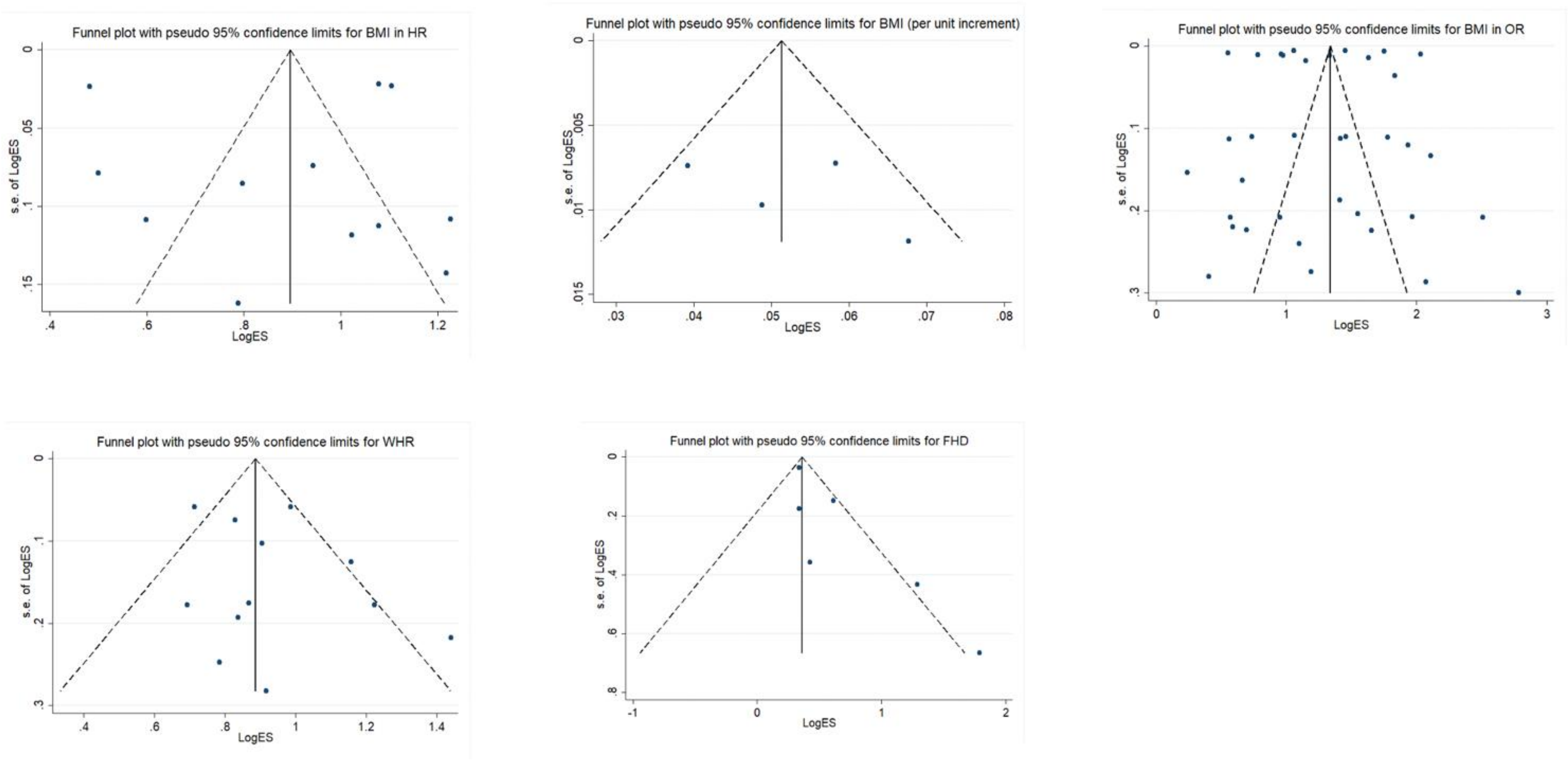

Figure S3a-c. Ethnic-specific optimal cut-points of (3a) BMI, (3b) WC and (3c) WHR for T2D (men/women)

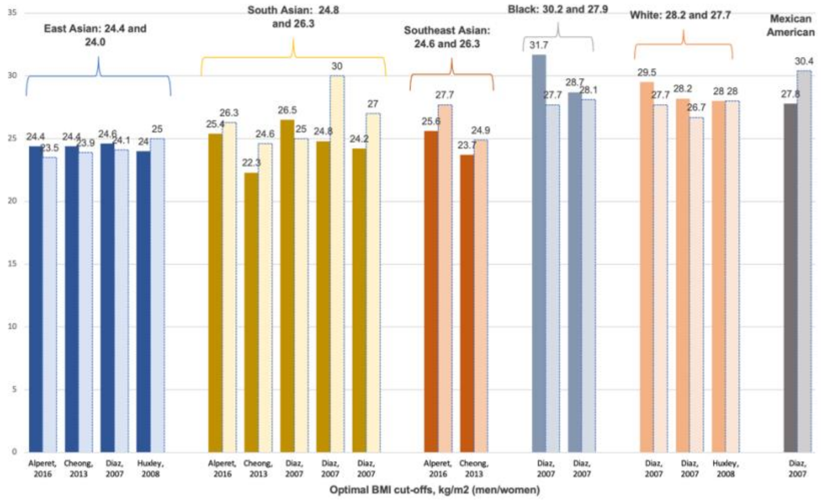

Figure S3a

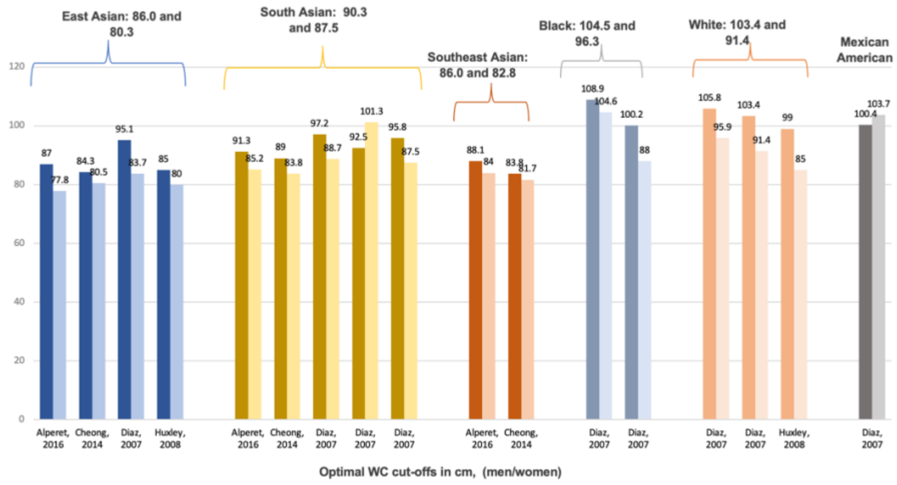

Figure S3b

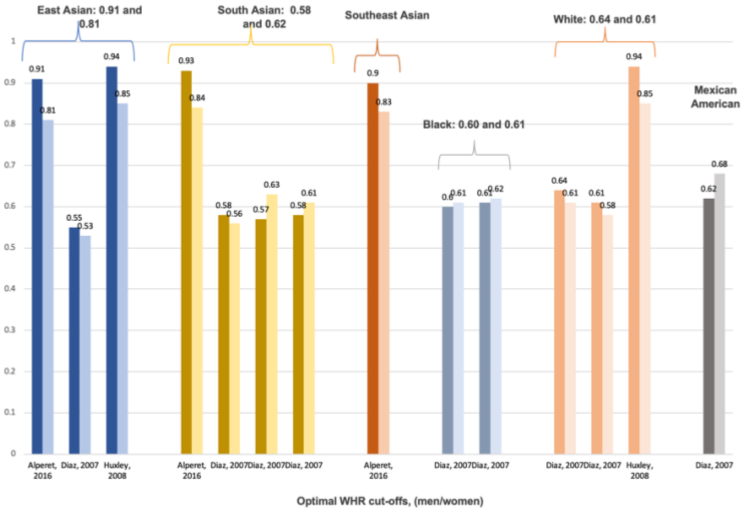

Figure S3c

Table S14. Trans-ethnic results of the effect of skinfold thickness, the ratio of subscapular to triceps, hand grip strength, WHHR, HC, trunk fat, ABSI, BAI on T2D.

Phenotype/Risk factor	Study	Results
**Skinfold thickness (indication of overall obesity)**	Mackay, 2010	OR (adj.) per SD change in log-transformed sum of skinfold thickness: 2.01 (95% CI 1.40-2.90) for NHW, 1.28 (95%CI 0.85-1.93) for Black/African American and 1.67 (1.18-2.38) for Hispanic group.
	Marshall, 1993	OR per 5mm increase in subscapular skinfold: 1.6 (95%ci 1.4-1.8) for NHW, 1.6 (95%CI 1.4-1.8) for Hispanic group.<br>OR per 5mm increase in triceps skinfold: 1.3 (95%CI 1.1-1.5) for NHW and 1.5 (95%CI 1.3-1.7) for Hispanic group.
**The ratio of subscapular to triceps (indication of the ratio of central to peripheral fat)**	Mackay, 2010	OR (adj.) per SD change in log-transformed measure:<br>2.34 (95% CI 1.51-3.62) for NHW, 2.78 (95% CI 1.65-4.70) for Black/African American, 1.12 (95% CI 0.75-1.67) for Hispanic group.
	Marshall, 1993	OR per 2-unit increase in subscapular to triceps ratio: 1.4 (95%CI 1.3-1.6) and 1.2 (95%CI 1.1-1.3) for Hispanic group.
**Hand grip strength**	Ntuk, 2017	OR for diabetes associated with hand grip strength for men: 1.5 (95%CI 1.4-1.6) for White, 1.6 (95%CI 1.202.0) for Black, 1.4 (1.1-1.7) for South Asian.<br>OR for diabetes associated with hand grip strength for women: 1.2 (95%CI 1.1-1.3) for White, 1.1 (95%CI 0.8-1.3) for Black, 1.6 (95%CI 1.1-2.0) for South Asian.
**WHHR (waist-to-hip-height ratio)**	Hardy, 2017b	HR per unit increase (z-score) in WHHR: 1.26 (95%CI 1.17-1.36) for White males, 1.28 (95%CI 1.17-1.40) for Black males, 1.61 (95%CI 1.47-1.77) for White females, 1.44 (95%CI 1.33-1.57) for Black females.
**HC (hip circumference)**	Mackay, 2010	OR (adj.) per SD change in log-transformed measure: 1.75 (95%ci 1.31-2.23) for NHW, 1.05 (95%CI 0.74-1.49) for Black/African American, 1.45 (95% CI 1.09-1.93) for Hispanic group.
	Steinbrecher, 2015	OR per SD increase in HC among men: 1.48 (1.38-1.61) for White, 1.28 (95%CI 1.17-1.41) for Native Hawaiian, 1.48 (95%CI 1.38-1.59) for Japanese American.<br>OR per SD increase in HC among men: 1.67 (95%CI 1.51-1.84) for White, 1.42 (95%CI 1.31-1.54) for Native Hawaiian, 1.69 (95%CI 1.56-1.83) for Japanese American.
**ABSI (a body shape index)**	Hardy, 2017a	OR per unit increase (z-score) in ABSI: 1.18 (95%CI 1.02-1.37) for White males, 1.34 (95%CI 1.13-1.60) for Black males, 1.47 (95%CI

		1.34-1.61) for White females, 1.44 (95%CI 1.31-1.58) for Black females.
	Hardy, 2017b	HR per unit increase (z-score) in ABSI: 1.00 (95%CI 0.94-1.06) for White males, 1.09 (95%CI 0.99-1.19) for Black males, 1.15 (95%CI 1.08-1.22) for White females, 1.19 (95%CI 1.09-1.29) for Black females.
**BAI (body adiposity index)**	Hardy, 2017a	OR per unit increase (z-score) in BAI: 1.99 (95%CI 1.72-2.30) for White males, 1.75 (95%CI 1.46-2.11) for Black males, 1.76 (95%CI 1.60-1.92) for White females, 1.28 (95%CI 1.17-1.41) for Black females.
	Hardy, 2017b	HR per unit increase (z-score) in BAI: 1.43 (95%CI 1.36-1.49) for White males, 1.35 (95%CI 1.25-1.45) for Black males, 1.54 (95%CI 1.47-1.62) for White females, 1.17 (95%CI 1.12-1.23) for Black females.
**Body height**	Jenum, 2005	OR per one-unit increase (cm) for women: 0.45 (95%CI 0.43-0.47) for White, 1.18 (95%CI 1.08-1.30) for South Asian.<br>OR per one-unit increase (cm) for men: 0.70 (95%CI 0.67-0.73) for White, 0.85 (95%CI 0.79-0.93) for South Asian.
**Trunk fat, trunk-to-leg ratio**	Luo, 2019	HR per unit increase in trunk fat: 1.60 (95%CI 1.51-1.69) for White, 1.40 (95%CI 1.25-1.58) for Black, 1.81 (95%CI 1.47-2.23) for Hispanic.<br>HR per unit increase in trunk-to-leg fat ratio: 1.52 (95%CI 1.44-1.60) for White, 1.69 (95%CI 1.49-2.20) for Black, 1.81 (95%CI 1.49-2.20) for Hispanic.
**Combination of anthropometric measures**	Steinbrecher, 2015	OR per SD increase in WC+BMI for men: 1.60 (95%CI 1.40-1.83) for White, 1.32 (95%CI 1.14-1.54) for Native Hawaiian, 1.59 (95%CI 1.42-1.77) for Japanese American.<br>OR per SD increase in WC+BMI for women: 1.31 (95%CI 1.12-1.53) for White, 1.23 (95%CI 1.08-1.40) for Native Hawaiian, 1.53 (95%CI 1.37-1.71) for Japanese American.<br><br>OR per SD increase in HC+BMI for men: 1.84 (95%CI 1.62-2.09) for White, 1.44 (95%CI 1.24-1.68) for Native Hawaiian, 1.68 (95%CI 1.51-1.86) for Japanese American.<br>OR per SD increase in HC+BMI for women: 1.99 (95%CI 1.66-2.38) for White, 1.51 (95%CI 1.30-1.76) for Native Hawaiian, 2.16 (95%CI 1.89-2.45) for Japanese American.<br><br>OR per SD increase in WHR+BMI for men: 1.70 (95%CI 1.55-1.85) for White, 1.37 (95%CI 1.24-1.51) for Native Hawaiian, 1.65 (95%CI 1.53-1.78) for Japanese American.

		OR per SD increase in WHR+BMI for women: 1.72 (95%CI 1.55-1.90) for White, 1.47 (95%CI 1.35-1.59) for Native Hawaiian, 1.81 (95%CI 1.67-1.96) for Japanese American.<br><br>OR per SD increase in WHTR+BMI for men: 1.47 (95%CI 1.28-1.69) for White, 1.30 (95%CI 1.12-1.52) for Native Hawaiian, 1.66 (95%CI 1.48-1.87) for Japanese American.<br>OR per SD increase in WHTR+BMI for women: 1.21 (95%CI 1.03-1.43) for White, 1.26 (95%CI 1.10-1.44) for Native Hawaiian, 1.54 (95%CI 1.37-1.73) for Japanese American.
	De Koning, 2010	OR per SD increase in WC+BMI: the stronger effect in Europeans and Aboriginals. Not significant among Black, Hispanic and South Asians.<br>OR per SD increase in WHR+BMI: the strongest effect among Aboriginals, followed by Europeans, Hispanic and South Asians. Not significant among Black ethnic group.

Table S15. Trans-ethnic results of the effect of women-health-associated factors on T2D.

Phenotype/Risk factor	Study	Results
**Index of parity**	Almahmeed, 2017	*Women of Chinese/East Asian* ethnicity (ref. 1): adj.HR 0.90 (95%CI 0.76-1.06) for 2 deliveries, 1.13 (95%CI 0.89-1.43) for 3-4 deliveries, 4.59 (95%CI 2.36-8.92) for ≥5 deliveries.<br><br>*Women of South Asian ethnicity* (ref.1): 0.93 (95%CI 0.81-1.08) for 2 deliveries, 1.03 (95%CI 0.87-1.22) for3-4 deliveries, 1.23 (95%CI 0.81-1.87) for ≥5 deliveries.<br><br>*Women of White ethnicity* (ref. 1): 0.89 (95%CI 0.85-0.93) for 2 deliveries, 1.13 (95%CI 1.08-1.18) for 3-4 deliveries, 1.52 (95%CI 1.39-1.66) for ≥5 deliveries.<br>P for interaction test=0.02.
**Birth weight**	Ryckman, 2014	Birth weight category (ref. 6-7.9 lbs).<br>*White:* OR 1.43 (95%CI 1.25-1.62) for <6lbs., 0.99 (95%CI 0.90-1.10) for 8-9.9 lbs., 1.07 (95%CI 0.88-1.31) for ≥10 lbs.

		Black: OR 1.31 (95%CI 1.04-1.64) for <6 lbs., 1.12 (95%CI 0.91-2.66) for 8-9.9 lbs., 1.44 (95%CI 0.95-2.19) for ≥10 lbs.<br><br>*Asian:* OR 0.87 (95%CI 0.53-2.02) for <6 lbs., 1.56 (95%CI 0.92-2.66) for 8-9.9 lbs., 2.77 (95%CI 0.91-8.43) for ≥10 lbs.<br><br>*Hispanic:* 1.39 (95%CI 0.96-2.02) for <6 lbs., 0.95 (95%CI 0.66-1.37) for 8-9.9 lbs., 0.43 (95%CI 0.13-1.38) for ≥ 10 lbs.
**Breastfeeding**	Mayer-Davis, 2008	*Black/African American women*: OR 0.65 (95%CI 0.24-1.77).<br>*Hispanic women*: OR 0.19 (95%CI 0.05-0.76).<br>*White women*: OR 0.18 (95%CI 0.07-0.49).<br>p-value 0.17 for interaction. Unadjusted model.
**Age at menarche**	Dreyfus, 2012	Adjusted OR: 1.41 (95%CI 1.05-1.89) for White women, 0.94 (95%CI 0.68-1.30) for Black/African American women (p=0.09 for interaction).<br>Adjusted HR: 1.43 (95%CI 1.08-1.89) for White women, 1.20 (95%CI 0.87-1.67) for Black/African American women (p=0.52 for interaction).

Figure S4. Ethnic-stratified meta-analysis results of the effect of FHD on T2D.

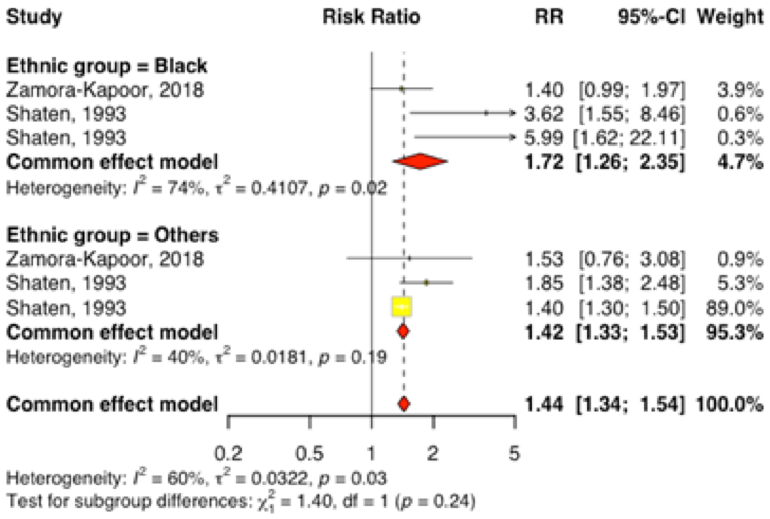

Table S16. Trans-ethnic results of the effect of family history of diabetes on T2D from studies not included in meta-analysis (Supplementary excel file)
